# Elongation during segmentation shows axial variability, low mitotic rates, and synchronized cell cycle domains in the crustacean, *Thamnocephalus platyurus*

**DOI:** 10.1186/s13227-020-0147-0

**Published:** 2020-01-18

**Authors:** Savvas J. Constantinou, Nicole Duan, Lisa M. Nagy, Ariel D. Chipman, Terri A. Williams

**Affiliations:** 1 Biology Department, Trinity College, Hartford, CT USA; 2 Department of Molecular and Cellular Biology, University of Arizona, Tucson, AZ 85721 USA; 3 The Department of Ecology, Evolution and Behavior, The Alexander Silberman Institute of Life Sciences, The Hebrew University of Jerusalem, Edmond J. Safra Campus, Givat Ram, 91904 Jerusalem, Israel; 4 Present Address: Department of Integrative Biology, Michigan State University, East Lansing, MI 48824 USA; 5 Present Address: Bioinformatics and Quantitative Biosciences, Georgia Institute of Technology, North Avenue, Atlanta, GA 30332 USA

**Keywords:** Arthropod, Segmentation, Growth zone, Mitosis, Wnt, EdU

## Abstract

**Background:**

Segmentation in arthropods typically occurs by sequential addition of segments from a posterior growth zone. However, the amount of tissue required for growth and the cell behaviors producing posterior elongation are sparsely documented.

**Results:**

Using precisely staged larvae of the crustacean, *Thamnocephalus platyurus*, we systematically examine cell division patterns and morphometric changes associated with posterior elongation during segmentation. We show that cell division occurs during normal elongation but that cells in the growth zone need only divide ~ 1.5 times to meet growth estimates; correspondingly, direct measures of cell division in the growth zone are low. Morphometric measurements of the growth zone and of newly formed segments suggest tagma-specific features of segment generation. Using methods for detecting two different phases in the cell cycle, we show distinct domains of synchronized cells in the posterior trunk. Borders of cell cycle domains correlate with domains of segmental gene expression, suggesting an intimate link between segment generation and cell cycle regulation.

**Conclusions:**

Emerging measures of cellular dynamics underlying posterior elongation already show a number of intriguing characteristics that may be widespread among sequentially segmenting arthropods and are likely a source of evolutionary variability. These characteristics include: the low rates of posterior mitosis, the apparently tight regulation of cell cycle at the growth zone/new segment border, and a correlation between changes in elongation and tagma boundaries.

## Background

Arthropods are the most diverse phylum on earth, and much of that diversity derives from the variability in their segmented body plan. The developmental mechanisms that produce segments have been extensively studied in the model organism, *Drosophila*. But *Drosophila* is atypical among arthropods because it establishes segments simultaneously, through progressive subdivision of the embryo [1]. By contrast, the vast majority of arthropods add their segments sequentially, from a posterior region termed the “growth zone”. These species elongate while adding segments, thus posing fundamental questions that do not apply to the model system *Drosophila*: How does elongation occur in the posterior? How are elongation and segmentation integrated [2]. While some mechanisms of elongation are known (e.g., teloblastic growth in malacostracan crustaceans [3]), surprisingly little is known about the range of cell behaviors (e.g., cell division or cell movement) responsible for elongation throughout arthropods.

Because most species elongate significantly during segmentation, classical concepts of posterior growth generally invoke mitosis, either in posterior stem cells or in a vaguely defined posterior region of proliferation [4–8]. Cell movement has also been assumed to play a role in elongation in cases where embryonic shape changes dramatically [7–10]—and is documented in the flour beetle, *Tribolium castaneum* [11–13]. The current descriptive data suggest a large degree of variability in how sequentially segmenting arthropod embryos grow (reviewed in [7, 14, 15]). That variability has led to the suggestion of replacing the term “growth zone” with “segment addition zone” (e.g., [16, 17]) or “undifferentiated zone” [15] as possible alternatives. Because the relative contribution of various cell processes—division, size or shape change, movement—to embryo elongation have only recently begun to be quantitatively and systematically examined, it is challenging to find an appropriate catch-all term for all arthropods.

In contrast to our lack of understanding of cellular mechanisms of elongation, the models of the gene regulatory networks that pattern segments in sequentially segmenting arthropods are being tested more broadly (reviewed in [14, 18–21]). In the posterior growth zone, *Wnt* signaling activates the transcription factor *caudal* (*cad*), which, through downstream genes, progressively subdivides the anterior growth zone and eventually specifies new segments [19, 22]. In some systems, posterior *Wnt* signaling is also thought to keep posterior cells in a pluripotent state, presumably dividing as needed and thus fueling elongation [22–25]. To fully understand segmental patterning and interpret function via knock-down/knock-out studies, we need a more detailed understanding of the cellular mechanisms underlying elongation and growth [14].

Our collaborating labs analyzed the changes in the growth zone during segmentation in three pancrustaceans to compare between species: including two insects, the beetle, *Tribolium castaneum* [12], and the milkweed bug, *Oncopeltus fasciatus* [25]; and the crustacean described here, *Thamnocephalus platyurus*. *Thamnocephalus,* commonly named fairy shrimp, belong to the same order as the brine shrimp, *Artemia*. Both are branchiopod crustaceans, a taxon more closely related to insects than are malacostracan crustaceans (e.g., *Parhyale hawaiensis* [26, 27]). *Thamnocephalus* live in temporary freshwater ponds [28] and their life cycle includes desiccation-resistant encysted eggs (giving rise to commercially available cysts, primarily for toxicology studies, e.g., [29]). After rehydration, cysts hatch as swimming larvae with three pairs of head appendages and an undifferentiated trunk. Sequential segment addition and progressive differentiation gradually produce the adult morphology of eleven limb-bearing thoracic segments and eight abdominal segments, the first two of which are fused to form the genital region [5, 30–32]. The highly anamorphic development of *Thamnocephalus*, as well as their phylogenetic position, makes them an interesting comparison to other arthropods and we have previously shown that there are numerous Wnts expressed in the posterior during segmentation [35]. In addition, Notch signaling, a known feature of posterior patterning in some arthropods also slows segment addition in *Thamnocephalus* [37].

Here, we examine in detail the morphometric changes and cell behaviors associated with segment addition in *Thamnocephalus.* We demonstrate that segments from the third thoracic segment arise at a constant rate. We characterize the growth zone and newest added segment during segment addition using morphometric measures. Changes in these measures occur at tagma boundaries. Despite expectations for mitosis to drive elongation, we demonstrate that mitosis in the growth zone is relatively rare; it contributes to elongation, but at lower rates than anticipated. These results corroborate those of Freeman [33], who counted cells and mitoses in the trunk of the first three instars of *Artemia* larvae and found more mitoses near the anterior than posterior trunk region. Examination of cells undergoing DNA synthesis reveals discrete domains of apparently synchronized cells in the anterior growth zone and newest segment. In *Thamnocephalus,* boundaries of cell cycling domains correlate precisely with *Wnt* and *cad* expression in the growth zone, suggesting direct regulation of these behaviors by the segmentation gene regulatory network.

## Results

### Segment addition and morphogenesis occur progressively in *Thamnocephalus* larvae

**Fig. 1:**
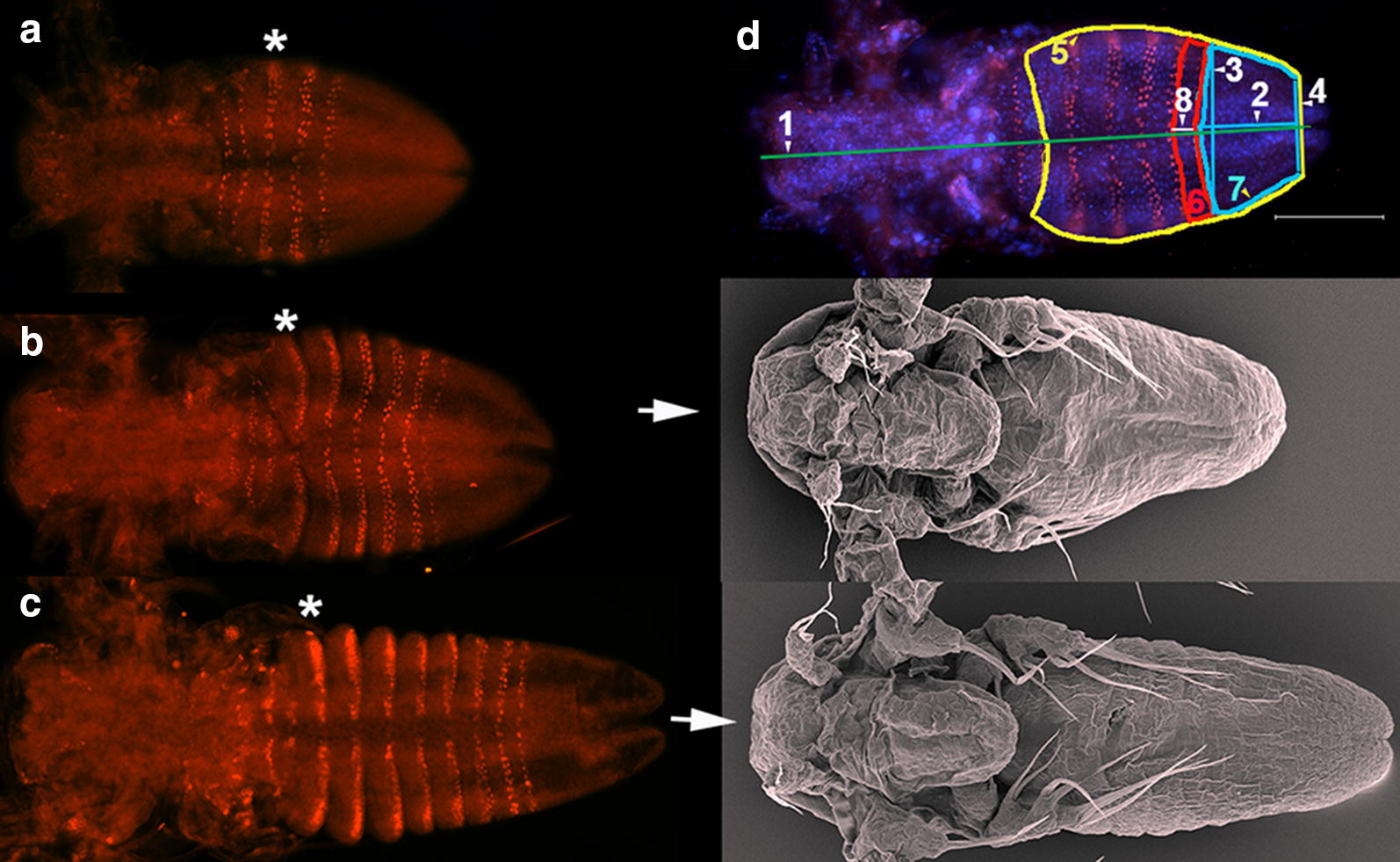
*Thamnocephalus* development and morphometric measures. **a**–**c** En protein staining in larvae with **a** three thoracic En stripes, **b** six thoracic En stripes, and **c** eight thoracic En stripes. Asterisks mark the first thoracic segment in each larva (the two stripes visible anterior to this are the first and second maxillary segments) and in **c** show the outpocketing of the segmental limb bud from the body wall. In **b**, **c** white arrow point to scanning electron micrographs of similarly staged larvae. **d** *Thamnocephalus* larva illustrating measurements used in this study (defined in “Materials and methods”): 1—body length, 2—growth zone length, 3—growth zone width “A” (width of newly added En stripe), 4—growth zone width “B”, 5—ventral trunk area, 6—ventral area of last segment, 7—ventral growth zone area, 8—last segment length. Note, the area measures are in color; length measures are given in white and denoted with an arrowhead. Scale bar = 100 μm. En expression (red). All larvae are shown with anterior to the left, ventral side up

*Thamnocephalus* hatches with three differentiated larval head appendages (first antennae, second antennae and mandibles, [34]). In addition, the first and second maxillae and on average three thoracic segments are already specified, as determined by the expression of a monoclonal antibody (En4F11) that recognizes the segment polarity protein, Engrailed (En). As larvae grow, segments are added gradually from the posterior growth zone (Fig. 1), with expression of En at the anterior of the growth zone indicating specification of a new segment. Segments mature gradually, so the trunk typically shows the progression of segmental development: segment patterning, segment morphogenesis, and limb morphogenesis (see [35]). As segments develop, epithelial changes at the intersegmental regions lead to bending of the epithelium and outpocketing of the ventral to ventrolateral surface (Fig. 1c, described by [36]). The initial outpocketing has a highly aligned row of cells that form its apical ridge. The entire ventrolateral outpocketing eventually forms the limb bud and will develop medial folds along its margin, producing the anlage of the adult limb branches before limb outgrowth [34, 35].

**Fig. 2:**
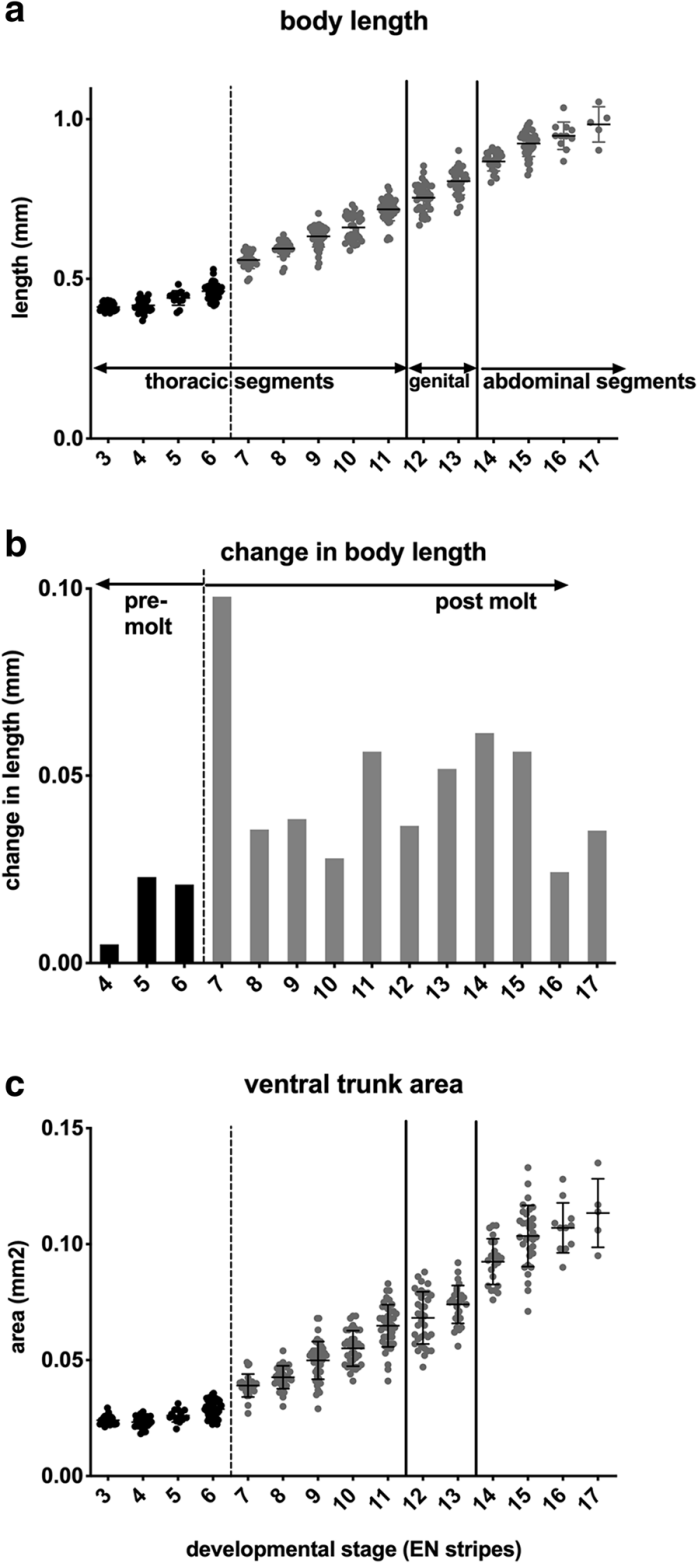
Elongation of the body at successive developmental stages in *Thamnocephalus*. **a** Body length plotted against developmental stage. The animals roughly double in length as the body segments are specified. **b** Percent change in body length plotted against developmental stage, demonstrating the impact of the first molt on change in body length. **c** Overall ventral area of the trunk increases at each stage (after four En stripes added). Black bars represent the thoracic segments added prior to the first molt (dashed line), subsequent thoracic segments are gray. Genital segments (modified abdominal segments 1 and 2) are marked by solid lines and followed by additional abdominal segments. Box and whisker representation of these data in Additional file 3. On average, 23 larvae per stage were scored for a total of 433 larvae, exact distribution of larvae in each hour and developmental stage included in Additional file 15

To characterize the rate of segment addition, we measured the number of segments, as indicated by En stripes, in 1 h intervals for staged cohorts of 20–30 larvae. Despite variability within each time point, we see a clear trend of linear segment addition (Additional file 1). This supports and extends an earlier dataset of segmentation rate produced under less controlled conditions [37]. Segments are added at an average rate slightly less than one segment per hour at 30 °C (0.7 segments/h or 1.4 h per segment). The regularity of segment addition is unaffected by either the first molt (~ 4 h post-hatching, see Additional file 2 for how first molt was determined) or the transitions between addition of thoracic (post-maxillary segments, 1–11), genital (12, 13), and abdominal segments (14–19, Additional file 1). Within 18 h at 30 °C, larvae add 14 segments, and the overall length of the body roughly doubles (Fig. 2a, Additional file 3). Despite the regular periodicity of segment addition, the change in body length at each stage varies, with an increase following the first molt (Fig. 2b). The overall ventral surface of the trunk also increases in both length and width at successive larval stages (Fig. 2c).

### The size of the growth zone varies during axial elongation and doubles in size to produce all segments

**Fig. 3:**
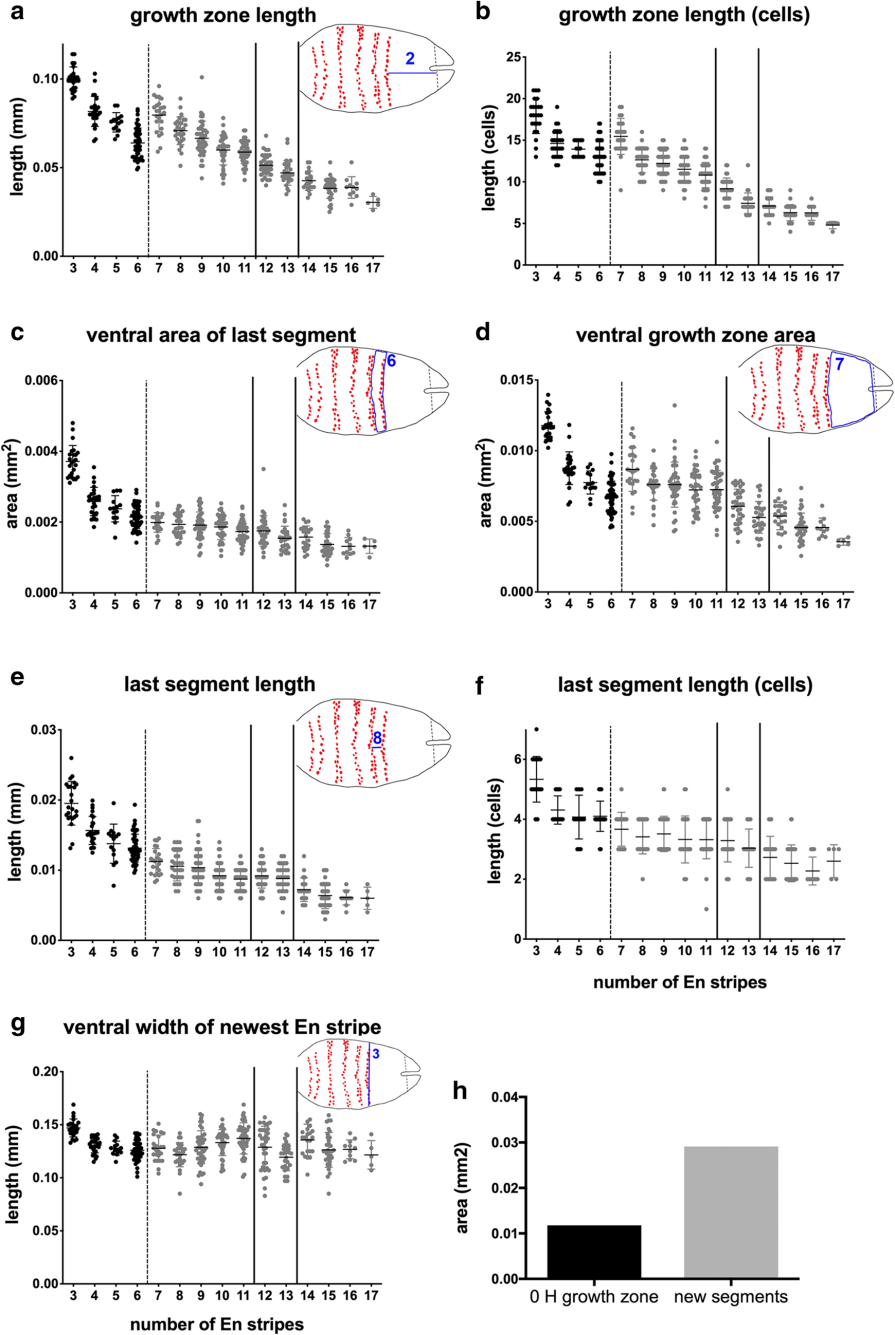
Change in growth zone dimensions in growing *Thamnocephalus* larvae. **a** Growth zone length decreases except after the first molt. This trend is the same when measured by counting cells (**b**). **c** The ventral area of the last added segment decreases in *Thamnocephalus*. **d** The ventral area of the growth zone decreases, except after the first molt. **e** The newest segments are longest during early stages. **f** When measured by counting cells, the length of the newest segment added mimics the linear dimension in **e**. **g** Unlike other dimensions, the width of the newly specified Engrailed stripe remains relatively constant during development (growth zone width “A” measure). **h** A comparison of the average size of the initial growth zone upon hatching (black column) *versus* the area required to make all additional segments (gray column), where the latter is calculated based on the sum of each newly added segment over the measured course of development. Trunk icon diagram measures represented in each panel and illustrate how ventral area was measured for these comparisons. Bar colors and lines, as in Fig. 2

To assess whether the growth zone itself changes over time and to estimate the growth occurring as segments are added, we measured several features in each stage (Fig. 1d). In general, most growth zone measures decrease as segments are added (Fig. 3, Additional file 4). Both the length and the ventral surface of the growth zone decrease over time. The exception to this trend occurs at the first molt, (between approximately 6 and 7 En stripes or around 3.75 h at 30 °C; Additional file 2; dotted lines Fig. 3). Post-molt, the growth zone increases in length (Fig. 3a, b; tagmata are separated in the graphs by solid lines; Additional file 3) and area (Fig. 3d), which is expected after release from the cuticle. Although the overall trend of a successively depleted growth zone matches the successive addition of segments, our analysis of another anostracan branchiopod, *Artemia*, shows that this is not the only possibility: in *Artemia,* the growth zone is not depleted over time but maintains its size through the addition of the first 9 En stripes (Additional file 4).

In addition to linear measures, we counted numbers of cells (nuclei) along our measured linear dimensions. Cell counts describe growth by the biological unit of cellular dimensions. For example, the smaller segments that are added posteriorly are only 2–3 cells long compared to about 4 cells long in the early segments added. The increase in cell number along the length of the growth zone at the molt is, on average, 2.5 cells.

**Fig. 4:**
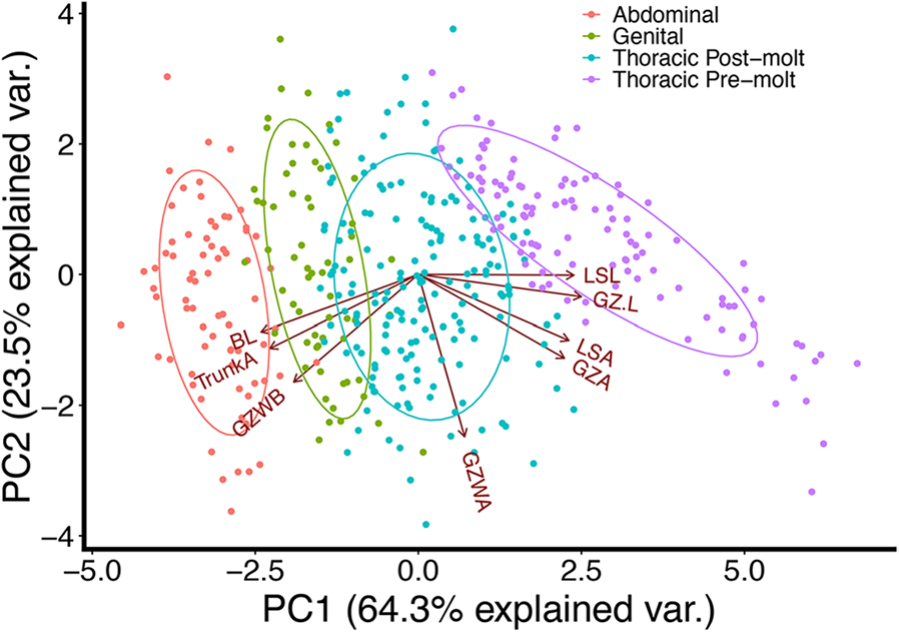
PCA biplot with tagma grouping. 423 individuals are plotted along PC1 and PC2 and grouped by tagma (in which the measures were made). PC1 explains 64% of the total variance in the data and separates individuals by tagma; a linear regression of PC1 on tagma indicates that “tagmata” are a good predictor of PC1 (adj *R*^2^ = 0.78; *p* < 0.001). Each tagma group is significantly different from one another (Type II MANOVA; *F*_9,1272_ = 103.06, *p* < 0.001). In addition, thoracic pre- and post-molt segments form clusters that are significantly different from all other tagma

To examine whether axial position was significant during segment addition, axial positions were split into four groups for statistical analysis, with measures assigned to tagma based on the axial position of the last added En stripe: En stripes 3–6 = thoracic (pre-molt); 7–11 = thoracic (post-molt;) 12–13 = genital; 14–17 = abdominal. We find that axial position is significant in most morphometric measurements, when individuals are grouped by tagmata and compared (Additional file 5). For example, each tagma forms segments from a successively smaller growth zone, whether measured by length (Fig. 3a, b) or area (Fig. 3d). By contrast, the one measure that remained notably steady between tagmata was the ‘growth zone width A’ measure, which is the width of the last En stripe (Fig. 3h). We further tested these trends by analyzing morphometric measurements using principal component analysis (PCA). PC1–PC3 explain 93.0% of the variation in the data and we found significant differences by tagmata (Fig. 4; Type II MANOVA; *F*_9,1272_ = 103.06, *p* < 0.001). PC1 explains 64.3% of the variance and separates by ‘tagma’; a linear regression of PC1 on tagma shows that “tagmata” are a good predictor of PC1 (adj *R*^2^ = 0.78; *p* < 0.001). Intriguingly, the thoracic segments added pre- and post-molt form groups that are as distinct as the other ‘true’ tagmata. While a linear regression of the number of segments (as a proxy for “axial position”) against PC1 also shows significance (since they are by definition highly correlated; Additional file 6), we point out that tagmata are likely are the relevant functional and evolutionary characters and thus it is notable that growth zone measures scale with changes in those characters.

During the time we tracked segment addition, approximately 14 segments were added. Body length increased about 140%, from 0.41 mm to 0.98 mm (Fig. 2a). The total ventral surface of the 14 added segments—when measured just as each is formed in successive stages—represents an area equal to 0.029 mm^2^. The area of the ventral surface of the initial (hatchling) growth zone is 0.0118 mm^2^ or only about 40% of the total ventral area ultimately needed to add all the segments (Fig. 3h). During segmentation, the growth zone shrinks (Fig. 3a, d), but even a completely depleted growth zone would only account for the addition of approximately the first four added segments. The growth zone needs to more than double to produce the material for new segments; it cannot account for all additional segments without some form of growth.

### The growth zone has few mitotic cells and shows little growth

**Fig. 5:**
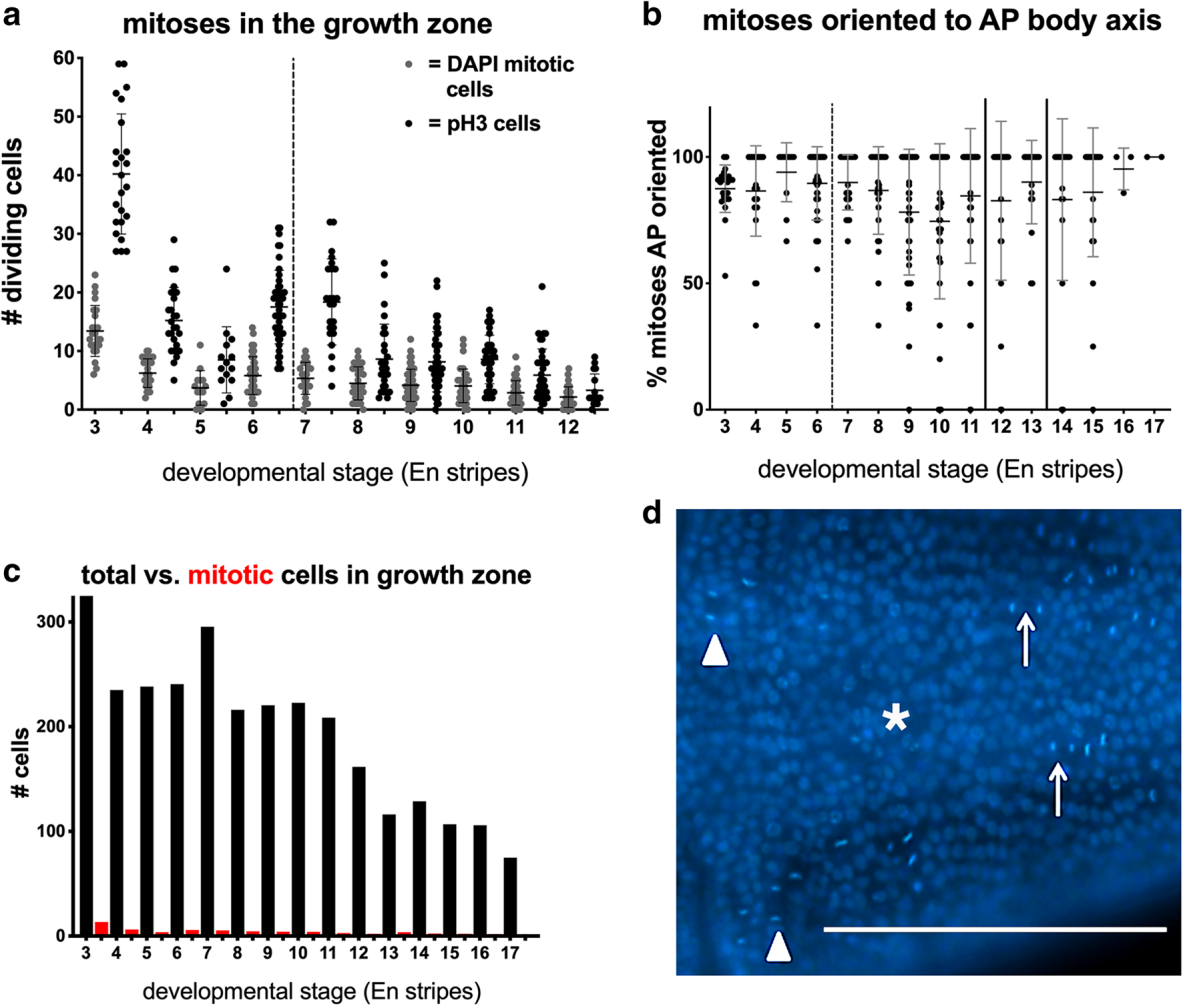
Mitosis in the growth zone of *Thamnocephalus*. **a** Scoring pH3-positive cells (black columns) in the growth zone captures consistently higher numbers of cells in M-phase compared to cells measured with nuclear staining (gray columns, Hoechst). Mitosis rates are highest just after hatching and increase prior to the first molt (dotted line). **b** Regardless of developmental stage, ~ 80% of the actively dividing cells (Hoechst) in the growth zone are oriented along the AP body axis. **c** Total calculated number of cells in the growth zone (black columns) compared to average number in mitosis (red) at successive developmental stages. (For comparison, the first red column is pH3 positive cells the second Hoechst. pH3 data were not collected after 12 h and the averages for the Hoechst scored mitotic figures drop to 1 and 2.) **d** Representative photo of AP-oriented cells in the GZ (arrows) in an early larva, although not stained with Engrailed, the approximate position of the last En stripe is indicated (asterisk). Note the medial–lateral oriented cells in the developing segments (arrowhead). Scale bar equals 100 µm

The larval epithelium is attached to the cuticle in *Thamnocephalus*, making significant bulk cell movements unlikely. Thus, to characterize growth in the growth zone, we focused on mitosis. We first counted mitosis by identifying cells clearly in metaphase, anaphase, or telophase using nuclear staining (Hoechst). The highest numbers of mitoses scored in this way were counted immediately following hatching, with an overall trend of fewer mitoses in the growth zone as segment addition continues (Fig. 5a, gray bars). Mitotic numbers increased slightly before and after the first molt (dotted line in Fig. 5a), but overall mitosis counts are low (ranging from about 2 to 13 cells). We also scored the orientation of the mitotic spindle and found that mitoses in the growth zone are oriented parallel to the anterior–posterior (AP) body axis. An average of 80% of all cells dividing in the growth zone are oriented in the AP direction, with as many as 90% in some larval stages (Fig. 5b). While mitotic cells in the growth zone are almost always oriented parallel to the AP body axis, mitoses in the newly specified segments are generally oriented transversely (Fig. 5d, not quantified).

To corroborate these measures of mitosis, we scored cells that express phosphorylated histone H3 (pH3) which is a common marker for mitosis [38]. Measures of pH3 labeling show stage-specific trends consistent with measures obtained by Hoechst (Fig. 5a, black bars; 2.4 × more on average). However, Hoechst and pH3 measures sometimes showed poor correlation within an individual (Additional files 7 and 8). While the pH3 signal is required for cells to enter anaphase [39], the stages of the cell cycle in which pH3 immunoreactivity can be detected vary between species [40]. In *Thamnocephalus*, immunoreactivity of pH3 fades before anaphase (data not shown). Thus, for any given specimen, cells scored in metaphase, anaphase, or telophase with Hoechst were not always a subset of those scored by pH3 (prophase/metaphase; Additional file 8) and single photographs of either Hoechst or pH3 used to represent typical mitoses may not represent average mitotic rates. Strikingly, even the greater numbers of cells in mitosis revealed by pH3 staining are low relative to the total number of growth zone cells (Fig. 5c).

We combined these direct measures of mitosis with our cell counts of the ventral surface of the growth zone to produce estimates of how much division might be needed for segment addition. Based on both direct cell counts of the length and width of the ventral surface of the growth zone and calculated cell counts of the area of the ventral surface of the growth zone area, the cells in the initial growth zone would need to divide about 1.5 times to produce enough cells to account for the addition of all the new segments (14) measured in this study (see Additional file 9). While this number is low, it is supported by our direct measures of mitosis compared to total growth zone cell numbers (Fig. 5c): mitotic cells only make up 1–4% of the cells in the growth zone. Consistent with this observation, the area of the ventral surface of the larval trunk increases over time (Fig. 2c) much more rapidly than the growth zone or last segment areas decrease, showing that the apparent growth of larvae is disproportionate in the already specified segments, and not in the growth zone per se.

### EdU incorporation reveals distinct domains of cell cycling

**Fig. 6:**
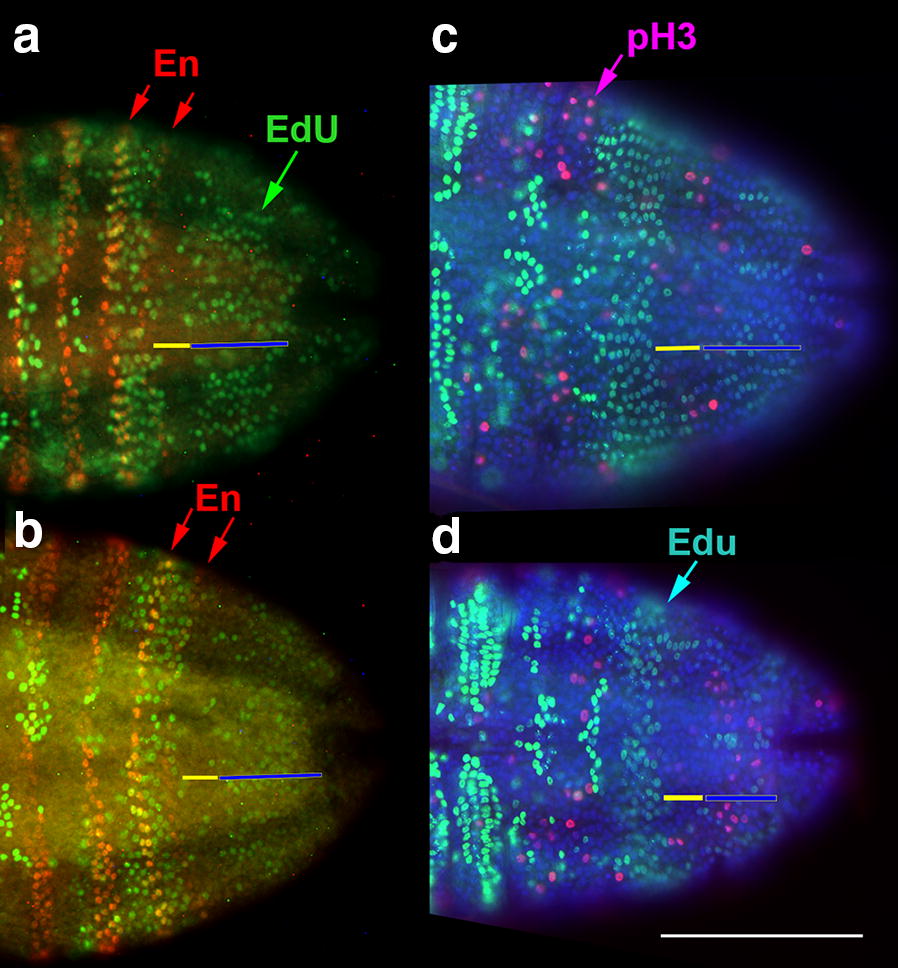
Cells synchronized in S phase in newest segment while the anterior growth zone has few cells in S phase. **a**, **b** After 30 min of exposure to EdU, a band of cells in S phase is visible (green) in the last added segment (red arrows indicate last two En stripes) in *Thamnocephalus*. This pattern is maintained throughout the early stages as seen in representative 1 h (**a**) and 2 h (**b**) larvae. The band lies almost entirely within the last segment after En segment specification. **c**, **d** In both 1 h (**c**) and 2 h (**d**) larvae, cells in the last added segment (EdU band, light green) do not show pH3 staining (pink) indicative of M-phase. Anterior growth zone is indicated by yellow bars; posterior growth, blue bars. Scale bars equal 100 μm

**Fig. 7:**
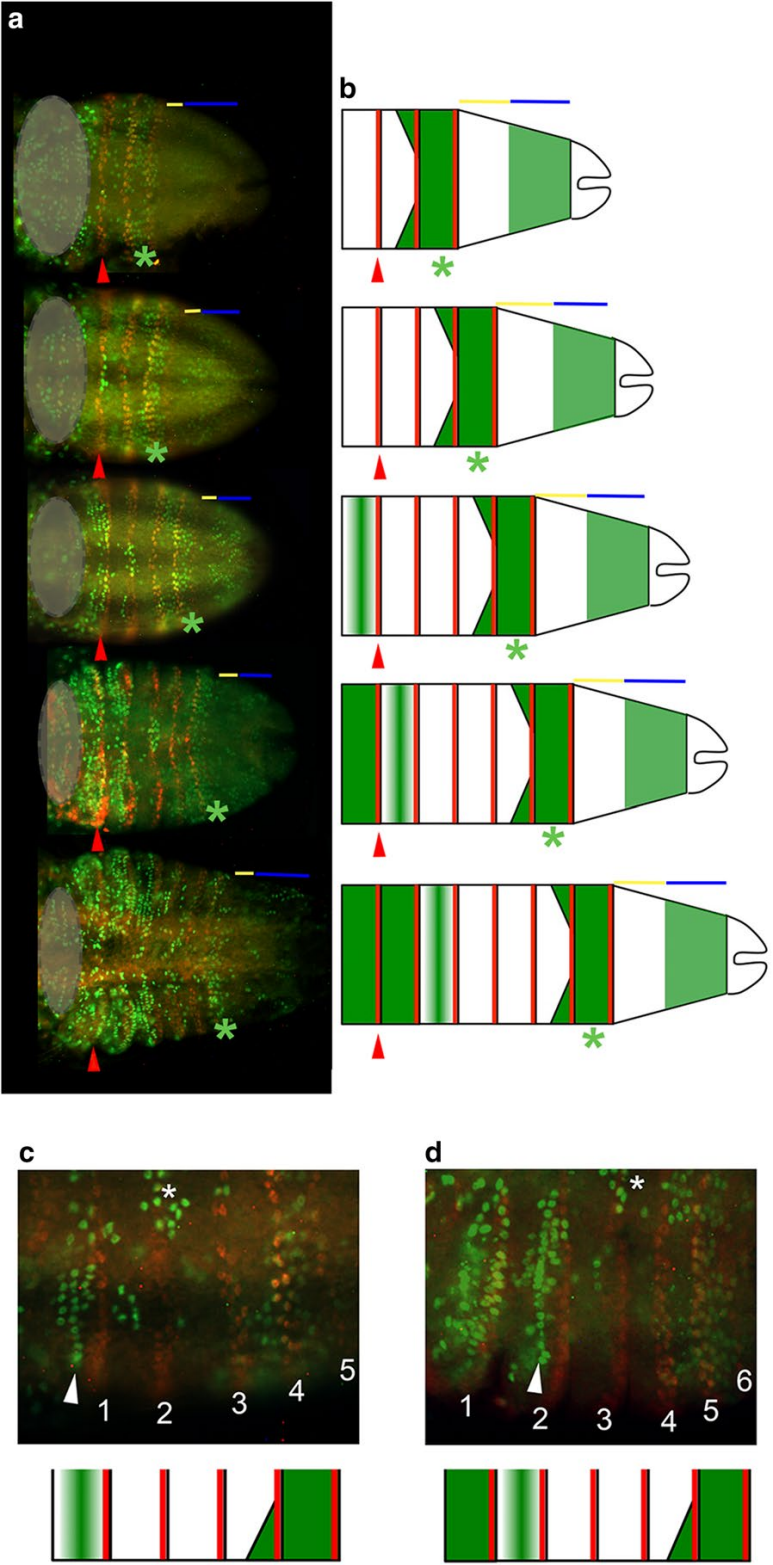
EdU incorporation in anterior segments shows stereotyped progression in early *Thamnocephalus* larvae. **a** Representative larvae with three to seven segments, oriented anterior left; the trunk is posterior (right) to the gray circle (which covers the head segments for clarity). **b** Diagrammatic representation of larvae highlighting the progression of EdU incorporation in the trunk. **a**, **b** In each stage, the first thoracic segment (red arrowhead) and the EdU band (green asterisk) are indicated. The anterior growth zone (yellow bars) is devoid of EdU, while the posterior growth zone (blue bars) has variable numbers of cells incorporating EdU. In the last added segment, all cells incorporate EdU (green asterisk), forming a band of EdU that sometimes extends into the lateral edges of the penultimate segment. The two segments anterior to this are devoid of EdU. Anterior still, segments begin to progress through S-phase, beginning as a discretely aligned row of cells at the apical ridge of the segment that then expands throughout the segment. **c**, **d** Higher magnification of a series of hemi-segments to illustrate progression of EdU incorporation in the trunk. Thoracic segments are numbered and the EdU incorporating cells aligned along the apical ridge are indicated (arrowhead). The neuroectoderm cycles through S phase a few segments anterior to the EdU band (asterisk). Both a specimen (top) and corresponding diagrammatic representation (bottom) are given

Mitotic scores in fixed animals give only a snapshot of cell cycle behavior and potentially underestimate rates of cell division. To capture a longer time-course of cell cycling, we exposed animals to 5-ethynyl-2′-deoxyuridine (EdU), a nucleotide analogue incorporated into cells during active DNA synthesis (S phase). A 30-min exposure to EdU before fixation labeled cells actively synthesizing DNA. This method revealed surprisingly stable domains of cell cycling in the larvae (Figs. 6 and 7).

#### The growth zone and newly added segment form three distinct EdU domains

In early larval stages analyzed in detail (0, 1, 2, 3, 4 h cohorts), we found a pattern of EdU incorporation that subdivides the growth zone into anterior and posterior domains: the posterior growth zone has randomly positioned cells undergoing S phase, while the anterior part of the growth zone mostly lacks cells in S phase (Fig. 6 Additional file 10). Note that a few S-phase cells can be found in the anterior growth zone. Just anterior to the growth zone, in the newest specified segment, all cells undergo S phase synchronously (all cells initiate DNA synthesis within a 30-min time window). That is, a band of EdU-expressing cells fills the last added segment, sometimes with additional, adjacent cells extending laterally into the penultimate segment (Fig. 6a, b).

Within all cohorts, these three domains are present and distinct. The two anterior domains—the EdU synchronous band and the EdU clear band—are easily identifiable. The most posterior domain, where apparently random cells undergo S phase, is more variable. In that region, there are three general classes of EdU incorporation: labeling in many growth zone cells (e.g., Fig. 6a), labeling in few growth zone cells (e.g., Fig. 6d), or in bilateral clusters of cells anterior to the telson. Furthermore, in the posterior growth zone, measures of mitosis (pH3) are low compared to cells in S phase, suggesting these cells are cycling at low and uncoordinated rates or have variable lengths of time in G_2_. By contrast, cells in the EdU band in the last segment appear synchronous. In specimens double-labeled with pH3 and EdU, pH3-positive cells are typically (but not always) excluded from this EdU domain, suggesting that cells within the domain are synchronizing their behavior at the anterior growth zone/newly specified segment boundary (Fig. 6c, d).

#### Segments in early larvae follow a stereotyped pattern of S phase as they develop

In contrast to the three stable domains of the growth zone region described above, we saw stage-specific patterns of S phase (identified through EdU incorporation) in the more anterior specified segments examined at different stage cohorts. Each segment undergoes a stereotyped pattern of S phase cycling as it develops (Fig. 7a, b): first, nearly all cells in the segment are in S phase (when the segment is first specified), then cells in S phase are localized to the lateral flanks, then S phase cells are concentrated in the neuroectoderm (not shown in Fig. 7), then S phase is initiated in cells at the apical ridge of the ventral outpocketing segment (in cells that express *Wnt1,* and other *Wnt* genes, just anterior to En [35]), finally, S phase spreads into other cells throughout the segment.

Thus, the overall, appearance at any larval stage depends on the number of segments specified. In 0-h animals, the two relatively small maxillary segments anterior to the thorax show high levels of EdU incorporation, although thoracic segments 1–3, which are already expressing segmentally iterated stripes of En, do not. As animals age (1–4 h post-hatching) and add more segments, the pattern of anterior segments undergoing S phase continues towards the posterior (Fig. 7).

### Domains of cell cycling in the growth zone correspond to boundaries of *Wnt* and *caudal* expression

**Fig. 8:**
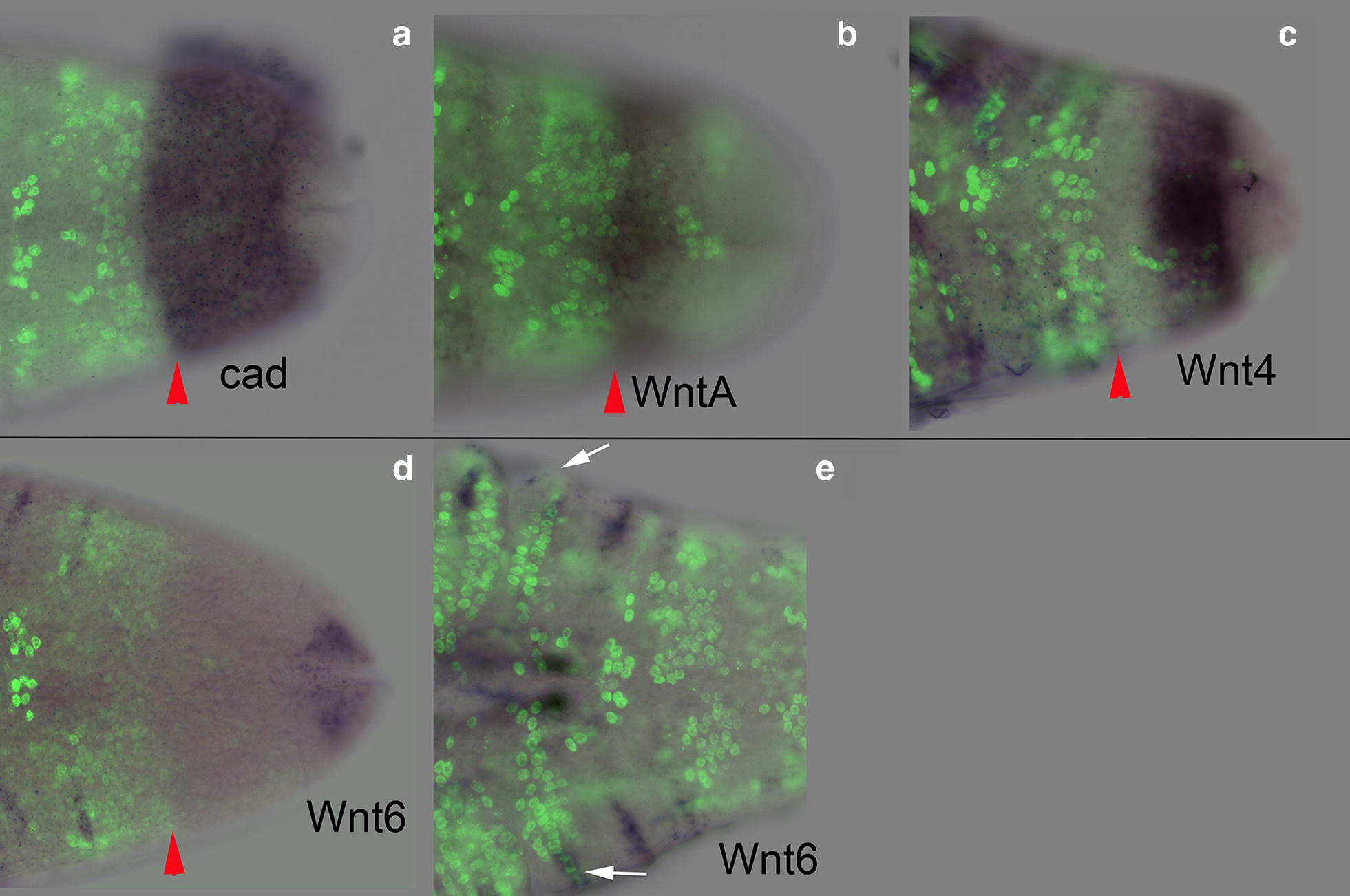
*Caudal* and *Wnt* gene expression maps directly to boundaries of EdU domains. Posterior of larvae showing both in situ expression domains and EdU incorporation. In each case, anterior is left and the posterior edge of the EdU band (red arrowhead) is denoted. **a** *Cad* expression extends throughout the entire growth zone and borders the telson, overlapping the posterior *Wnt4* and *WntA* expression. **b** Posterior *WntA* expression is mainly in the anterior growth zone, where there are very few to noEdU positive cells. The anterior border of *cad* (**a**) and *WntA* (**b**) both flank the posterior edge of the synchronized EdU band in the newest specified segment. **c** Posterior *Wnt4* expression excludes the band with rare EdU staining and overlaps with the unsynchronized EdU region in the posterior growth zone. *Wnt4* also appears to have a concentration gradient from posterior border towards anterior border. The anterior border of *Wnt4* expression meets the posterior border of *WntA* expression. **d** *Wnt6* is expressed in the telson and **e** in the cells that form the apical ridge of the limb buds, which also show EdU expression (white arrows)

We analyzed expression of *caudal* and *Wnt* genes relative to EdU incorporation in the posterior, looking specifically at three *Wnts* shown to have staggered expression in the growth zone: *Wnt6*, *WntA*, and *Wnt4* [35]. Expression of *cad* is non-graded and extends throughout the growth zone to the border with the telson (Fig. 8a). *WntA* is expressed exclusively in the anterior and *Wnt4* is expressed exclusively in the posterior, and show graded expression [35] (Additional file 11). Strikingly, the domains of Wnt expression map to the domains of EdU incorporation in the growth zone: *WntA* expression in the anterior corresponds to cells lacking EdU incorporation (Fig. 8b) and *Wnt4* in the posterior corresponds to cells with scattered EdU incorporation (Fig. 8c). More anteriorly, the last two stripes of *Wnt4* expression, i.e., the most recently formed, appear to flank the band of coordinated EdU positive cells (Fig. 8c). The anterior border of both *cad* and *WntA* also coincides with the posterior border of the EdU domain in the newest segment. Posterior *Wnt6* expression is restricted to the telson, that is, behind the region of relatively dense cells that make up the posterior growth zone (Fig. 8d). Interestingly, limb bud cells that form the apical ridge and express *Wnt6* are also those that show the early apical EdU incorporation (Fig. 8e).

## Discussion

### Is there growth in the “growth zone”?

In sequentially segmenting arthropods, axial elongation appears coupled to segmentation in a way that supports the assumption that posterior segmentation is linked to posterior growth. This assumption has been both explicitly recognized [7, 14] and challenged [16], leading to the designation of the posterior as a “segment addition region” rather than a “growth zone”. Furthermore, it is clear in some insects that classical views of a proliferative posterior growth zone are inadequate to explain changes in embryo shape that can accompany segmentation during embryogenesis, and that cell movement plays a significant role in some cases. These cell movements can drive rapid elongation, as live imaging and clonal analysis have begun to show (for example, *Drosophila* [41]; *Tribolium* [12, 42]). In addition, a number of arthropod species show conserved expression of Toll receptors during elongation, with a functional role in normal elongation in both the flour beetle and spider [43]. Nonetheless, for the vast array of arthropods, the phenomena responsible for posterior elongation remain unknown and understudied, especially compared with the exploration of patterning genes regulating segmentation. The general morphometric changes accompanying elongation have been studied systematically in two insects—*Tribolium* [12, 44] and *Oncopeltus* [25]—both of which show a limited amount of growth. Here, we used careful staging to track growth in larvae of the crustacean *Thamnocephalus*, which appear to have a more obvious amount of posterior growth since they add most of their segments post-hatching. Growth could be by a posterior zone of high levels of mitosis, as is assumed for a classical growth zone [45].

Matching the expectation of growth, we documented a ~ 140% increase in body length during segment addition in *Thamnocephalus*. However, systematic examination of mitosis in the growth zone itself revealed a low percentage of cells in mitosis. We estimated that this low level of mitosis if sufficient) to provide enough tissue to form the new segments measured. These results highlight the misleading effect of including overall embryo/larval elongation when analyzing the role of the growth zone in forming new tissue for adding segments. Indeed, in a related anostracan, *Artemia*, Freeman [33] found the same general pattern in the trunk using morphological landmarks: more cells were in mitosis in the anterior trunk region than the posterior. In the few species in which mitosis has been examined during sequential segmentation [25, 44–46]; this study), mitosis in the already specified segments is extensive and no doubt contributes greatly to overall elongation. It is becoming clear that this overall elongation along the body leads to a false expectation of high mitosis in the growth zone and at the same time potentially obscures a low but real amount of posterior growth.

Interestingly, our estimates of growth in *Thamnocephalus* parallel our findings in insects: in *Oncopeltus*, growth zone mitoses were few and their localization revealed only by averaging over a number of staged embryos [25]; in *Tribolium*, clones of cells labeled in the blastoderm divided 2.4 times on average prior to germband elongation [12]. Our estimates for *Thamnocephalus* also parallel zebrafish data in which progenitor cells divide only one time after the presomitic mesoderm is established [47]. In summary, despite a measurable amount of increased area to account for the addition of new segments, the predicted amount of cell division needed to make the additional tissue is low and is corroborated by the low counts of mitoses based on direct measures of cells in the growth zone.

### Synchronized cell cycle domains map to boundaries of segmental gene expression

The most surprising feature of trying to quantify cell cycling in the growth zone in *Thamnocephalus* arose from exposing larvae to a nucleotide analogue (EdU) to visualize cells in S phase. This unexpectedly revealed distinct S phase domains, demonstrating a kind of spatial coordination in cell cycling not captured by examining mitosis alone. We found stable cell cycle domains at the anterior growth zone/newly added segment boundary. The best-known cell cycle domains are the mitotic domains in the embryos of flys: *Drosophila, Calliphora,* and *Musca* [48–50]. Among other arthropods, we do not know of a comparable case of highly synchronized cell cycle domains in the growth zone per se. Although not apparently as tightly synchronized, Auman et al. [25] found a similar regionalization of cell division in the growth zone of *Oncopeltus:* a region of low cell division in the anterior of the growth zone, and high cell division in the posterior. It is interesting to speculate whether, in these cases, the anterior growth zone is the region of segment pre-patterning and thus cell are not cycling. By contrast, examination of *Tribolium* using EdU exposure showed no apparent regionally distinct incorporation within the growth zone [44].

To interpret the fixed patterns of S phase domains in *Thamnocephalus*, we trace cell domains mapped to analogous positions in carefully staged larvae, leading to a hypothesized sequence of cell behaviors. Cells in the very posterior growth zone undergo low levels of uncoordinated cycling. Then, as they reach the anterior growth zone, they are coordinated and synchronized, perhaps by a cell cycle arrest. After they are newly specified into a segment, all cells undergo S phase synchronously. This entire progression of cell cycling is strikingly similar to that found in zebrafish somitogenesis. In zebrafish, progenitor cells first cycle in the posterior, then arrest in S/G2 as they transit the presomitic mesoderm to form a somite, then begin to cycle again due to upregulation of *cdc25* after somite formation [47]. Compartmentalized expression of *cdc25* in the tailbud is required for both extension of the body during somitogenesis and normal differentiation of posterior progenitor cells. We have begun to characterize the cdc25 (*string*) homolog as well as other regulators of cell cycle in *Thamnocephalus* (Duan and Williams, in prep).

We compared the domains of cells in S phase in *Thamnocephalus* with expression of genes known to regulate posterior segmentation and found that boundaries of gene expression map to boundaries of cell cycling. Both *cad* and some Wnts (mainly Wnt1 and Wnt8) are known to function in sequential segmentation in a number of arthropods by maintaining the growth zone and have been hypothesized to maintain cells in a proliferative state [22–24, 51]. A number of arthropods show expression of multiple Wnts in the growth zone (the spider *Parasteatoda tepidariorum* [16], the centipede *Strigamia maritima* [52], the millipede *Glomeris marginata* [53, 54], *Tribolium* [16, 55]), although in some cases it is difficult to infer their relative expression patterns and whether, like *Thamnocephalus*, the growth zone is divided by domains of distinct *Wnt* expression. Nonetheless, in all arthropods examined there are distinct regulatory signals in the anterior and posterior growth zone, with expression of *Wnt/cad* commonly in the posterior and pair-rule and or *Notch* pathway genes in the anterior growth zone [24, 25, 56]. Where it has been examined, Wnt/cad signaling regulates the genes of the anterior growth zone [23, 24, 57–59]. Our finding of anterior and posterior regionalization of cell behaviors in the growth zone that map to segmental gene expression is similar to what we found in *Oncopeltus:* the region of low cell division in the anterior of the growth zone is coincident with striped *even*-*skipped (eve)* and *Delta* expression, *versus* high cell division in the posterior coincident with *cad* and broad *eve* expression [25].

### Cell division in the *Thamnocephalus* growth zone is oriented in the anterior/posterior body axis

We found that almost all mitoses are oriented along the AP body axis in the growth zone of *Thamnocephalus*. AP-oriented mitoses can bias growth, impacting elongation via cell division, as da Silva and Vincent [60] demonstrate for *Drosophila* germband elongation. Whether it is important for elongation in other arthropods is unclear. It has also been described in *Artemia* by Freeman [33], who found, as we do, AP orientation in posterior cells but oblique and transverse orientation within segmented regions. It has also been described in malacostracan crustaceans, where two rounds of AP-oriented cell division in cells budded from the posterior teloblasts establish four rows of cells that form the initial segment anlage [61, 62]. Given the low rates of mitosis used by *Thamnocephalus*, it is unclear what function oriented mitosis might have on elongation or indeed whether it has any function at all and is instead a passive result of tissue-level mechanics. There could be other functions for oriented cell division, e.g., the efficient addition of new segments could be improved by orderly cell arrays, or precise molecular gradients may require cells in a particular orientation. Disrupting regulators of planar cell polarity in the growth zone epithelium could shed light on these potential functions.

### Changes in the growth zone are linked to different body tagmata

We document that the growth zone shrinks over time in *Thamnocephalus*: the posterior field of cells is depleted as segments are added. However, this decrease is not simply monotonic, but varies by the particular tagma in which segments are being added: the dimensions of the growth zone as well as the newest segmental anlage are statistically smaller when generating abdominal *versus* thoracic segments. This correlation is intriguing. It is known in vertebrates that extension of the embryo, while a continuous process, relies on different cell populations when forming the trunk versus tail [63]. The switch from trunk to tail is specifically regulated and mutants in *growth/differentiation factor 11 (Gdf11)* can lengthen the trunk by extending onset of the switch [64, 65]. While arthropod segmentation is phenomenologically quite different from vertebrates, relying on the subdivision of an epithelial sheet versus specification of motile, mesenchymal cells, we find it intriguing that our measures of the growth zone correlate with tagma boundaries. This may suggest that, in arthropods, very early segmental anlage are integrating different patterning signals along the body axis, and may similarly show some switch in cellular behaviors involved with early segment formation in different tagma.

The morphometric correlations with tagma do not have a corresponding temporal variation in *Thamnocephalus*: the rate of segment addition is constant. This is consistent with the other crustacean in which it has been measured, *Artemia* [37, 66], *Oncopeltus*, an insect that only adds abdominal segments sequentially [25], and the centipede, *Strigamia* [67]. By contrast, we showed that, in *Tribolium*, segmentation rate varies at the boundary between thorax and abdomen and correlates with a change in cell movement [12]. We hypothesized that the slowing of segment addition prior to the rapid addition of abdominal segments was necessary for the extreme cell movements that accompany abdominal segmentation. Sampling additional species, where both thoracic and abdominal segments are added sequentially, would increase our understanding of these phenomena, particularly how segmentation rate may change at axial position boundaries.

### Cell cycle domains in anterior segments

Examining EdU incorporation throughout the body in any arbitrary specimen shows a large number of cycling cells. At first glance these patterns of EdU incorporation appear somewhat random and widespread, but strikingly regular patterns of incorporation emerge from comparisons of precisely staged larvae. During early development, we see a progression of cells undergoing S phase from anterior to posterior in newly specified segments. This suggests a regular progression of cell cycling coupled to the visibly regular progression of morphogenesis in the specified segments [34, 35]. One of the first morphogenetic events in the segments is the ventral outpocketing of the limb bud. Freeman et al. [36] argue that greater cell mitosis in the limb bud anlage (compared to the intervening arthropodial membrane region) are required for the epithelial bending that generates this initial out-pocketed limb bud in *Artemia*. Thus, the synchronization of cell cycle in the early segmental anlage in *Thamnocephalus* may be used to accommodate or drive the subsequent morphogenesis of the limb bud.

Intriguingly, the pattern of EdU incorporation we describe in *Thamnocephalus* bears a striking resemblance to the domains of pH3 expressing cells in the wasp *Nasonia,* that similarly appear to progress from anterior to posterior during embryonic segmentation of successively older embryos [46]. Rosenberg et al. [46] document a series of mitotic domains lying exclusively between segmental *eve* stripes (at least in early embryonic stages). Interestingly, Foe [48] found that the boundaries of mitotic domains in *Drosophila* also corresponded to segmental boundaries (En stripes). Thus, the cell cycle domains in these three species are tied to segmental boundaries. This kind of domain-specific, timed cell cycling, bespeaks a tightly controlled integration of cell division and segment patterning. The presence of this phenomenon in distantly related arthropods begs for comparative analysis among other arthropod groups to determine if this cell behavior is an ancestral or derived trait.

## Conclusions

**Fig. 9:**
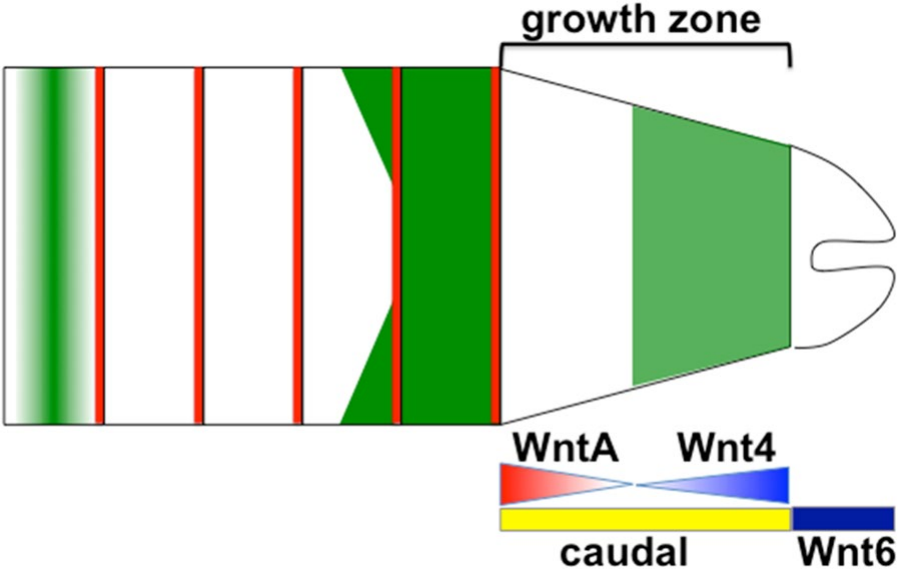
Diagram of growth zone in *Thamnocephalus*. The *Thamnocephalus* growth zone is divided into anterior and posterior regions based on cell behaviors and gene expression. The posterior domain corresponds to *Wnt4* expression (blue gradient); cell cycling in this region is present but low. Although mitosis in the posterior growth zone is not temporally or spatially synchronized, all mitosis in this domain is restricted in anterior–posterior orientation. The anterior growth zone corresponds to *WntA* expression (red gradient) and lacks cells in S phase. Cells in this region are possibly arrested either in early S phase or at the entry from G1 to S phase, since immediately after the anterior growth zone cells enter S phase again in the newest specified segment (dark green in last added segment). The synchronized S phase and subsequent mitoses in the segments generate the bulk of the visible elongation of the larvae. *Wnt6* expression (dark blue bar) is in the telson, posterior to the growth zone while *caudal* expression (yellow bar) is throughout the growth zone. S phase domains in green, En-expressing cells in red

In *Thamnocephalus,* we extend and confirm that segments are added at a constant rate. We find that the growth zone is depleted over time (shrinking cell field) while being partially replenished by cell division. The amount of cell division in the growth zone is low and the rate of cell cycling appears to be slower in the growth zone than in the newly specified segments. Cell division within the growth zone is aligned along the AP body axis although the impact of this on elongation of the body is predicted to be small relative to the increase in length caused by the rapid growth of segments once they are specified. The growth zone has two distinct domains (Fig. 9): a posterior *Wnt4* expressing region that has some cells undergoing S phase and M-phase and an anterior *WntA* expressing region that has no cells in S phase. Once a segment is specified, the cells of that segment enter S phase in a synchronous fashion. Newly specified segments then undergo a patterned sequence of entering S phase, starting with neuro-ectoderm, then the segmental apical ridge, before spreading broadly throughout the segment, forming an AP pattern of cell cycling along the body axis. While these growth zone features are stable in the early stages measured, other growth zone features change in association with the tagma in which segments are produced (e.g, linear dimensions). These kinds of cellular dynamics are only beginning to be measured in other species and yet already show a number of intriguing characteristics that may be more widespread among sequentially segmenting arthropods. First, we find surprisingly low amounts of posterior mitosis. We argue this mitosis contributes to normal elongation. This appears to be true, even for a number species that also use cell movement to elongate [12, 44]. What is clear is that, except for malacostracans, no arthropods show a narrow zone of dedicated proliferative cells in the posterior growth zone that would be similar to what has been documented in leeches or some polychaetes [68, 69]. So mitosis is occurring although at least in some species focused in the posterior region in the growth zone, presumably since the anterior region is where the segmental patterning is being finalized. In the anterior growth zone, we find the apparently tight regulation of cell cycle at the growth zone/new segment border, seen in the synchronization of cell cycling. Finally, we find the correlation between changes in the growth zone and tagma boundaries suggesting the importance of axial position, even at the formation of the earliest segmental anlage. These characters are likely a source of evolutionary variability underlying the segmentation process and our present choice of arthropod models may not be widely representative of the diversity of cell behaviors that underpin posterior elongation.

## Materials and methods

### Thamnocephalus *culture and fixation*

*Thamnocephalus* cysts (MicroBioTests Inc, Belgium) were hatched in 1:8 EPA medium:distilled water solution (EPA medium—0.0537 mM KCl, 1.148 mM NaHCO_3_, 0.503 mM MgSO_4_, and 0.441 mM CaSO_4_) at pH 7.0 and ~ 27 °C under a full spectrum aquarium lamp (T8 Ultrasun, ZooMed). For precisely staged animals, all hatchlings were collected from the tank every 15 min, raised at 30 °C under fluorescent light (~ 3500 lx) in a Precision 818 incubator. Animals were reared in 6-well cell culture dishes (~ 5 mL fluid per well; < 30 specimens per well) and fed 1 µL of food at time of collection. 4–18H animals received an additional 1 µL of food after a 60% water change at the midpoint of their rearing while 0–3 h animals were not fed since they are utilizing yolk reserves. Food consisted of a solution of yeast and commercially available fry food (Hikari First Bites) made fresh each day in 1:8 EPA medium. Animals were fixed for 30 min on ice in 9% formaldehyde/fix buffer (phosphate buffered saline supplemented with 70 mM EGTA) and then dehydrated to 100% methanol in a series of washes (2–3 min at 25%, 50%, and 75% methanol). Fixed larvae were stored at 0 °C in 100% methanol.

### *Artemia* culture and fixation

*Artemia* were raised in a 2.5 gallon tank at 25 °C, 30–35 ppt salinity using artificial sea salts, with continuous aeration and continuous full spectrum light. Newly hatched larvae were collected in timed intervals and were fed a mixture of yeast and algae (see above). Animals were fixed as *Thamnocephalus* (above) but with the addition of 0.1% Triton to the buffer.

### Immunohistochemistry

Immunohistochemistry protocols follow [70]. We visualized En using En4F11 (gift from N. Patel) and dividing cells using pH3 (anti-phospho-Histone H3 (Ser10) Antibody; Millipore) at 1 µg/mL. Specimens were counterstained with Hoechst, mounted in 80% glycerol supplemented with 0.2 M TRIS buffer and 0.024 M *n*-propyl gallate using clay feet on coverslips to prevent distortion, and photographed on a Nikon E600 Ellipse epifluorescence microscope and a Spot Insight QE digital camera (Diagnostic Instruments, Sterling Heights, MI, USA) and Spot Advanced software.

### EdU exposures and antibody or in situ doubles

Animals were exposed to 0.6 mM EdU for either 15 or 30 min just prior to fixation. EdU was visualized through the Click-iT^®^ EdU Alexa Fluor^®^ 488 Imaging Kit (Thermo Fisher Scientific) as described in the manufacturer’s manual with a final concentration of 1 µM sodium azide. For pH3 doubles, pH3 was visualized as above. Specimens were counterstained with Hoechst and mounted in 80% glycerol. Photographs were taken as above. For in situ/EdU doubles, animals exposed to EdU 30 min prior to fixation first underwent in situ hybridization for *caudal* and *Wnt4, WntA, Wnt6* as described previously [35]. After washing out the NBT/BCIP developing solution, animals were washed in 0.1% PBTriton, and processed through the Click-It reaction, as above.

### Molting

Individual animals were collected at hatching (*t* = 0) and allowed to swim freely in 1 mL of pond water in a 24-well plate (Falcon). The timing of the first molt was determined by observing single specimens under a dissecting scope every 5 min. The exuvia shed at the molt was visible. Immediately following the molt, the animals also displayed a characteristic behavior: individuals stayed at the bottom of the well and combed the setae on the antennal exopod by repeatedly pulling them between the mandible and coxal masticatory spine. After the first molt, the posterior trunk of the animal was elongated compared to the bean shaped trunk before the first molt (Fig. 1) which is reported for other branchiopods [71]. The setae on the coxal masticatory spine become branched, resembling a bottle-brush, compared to the non-setulated setae before the first molt (Additional file 2).

### Measured and calculated growth zone dimensions

All measurements were made directly on the photographs within the Spot software except number of mitotic cells in the growth zone which were counted in preparations under the microscope. Growth zone measures were confined to 2D projections of the ventral surface. We recognize that some information may be lost in projecting a three-dimensional surface onto two dimensions for measurement. Several properties of the branchiopod larvae suggest this approach nonetheless provides a valuable estimation of how the growth zone changes over time. First, the growth zone region does not differ materially between dorsal and ventral (Additional file 12). Second, the epidermis is a single layer with nuclei quite easy to see (Additional file 13) and developing branchiopod larvae have an extensive hemocoel beneath that single cell-layered epidermis [3] separating the epidermal nuclei from other tissues.

Measures were defined as follows:

Engrailed stripes (En): The number of En stripes posterior to the maxillary stripes. To be scored, the En stripe must extend from the lateral edge of the animal and connect across the ventral surface forming a complete line (i.e., the presence of few, scattered En-expressing cells was not scored as a new segment).

(Following numbers correspond to Fig. 1d, shown in detail in Additional file 14, with sample numbers for each stage in Additional file 15).

1. Body length (BL): measurement from the most anterior head region to anus through the midline.
2. Growth zone length (GZ length/cells): the growth zone length is measured at the midline from just posterior to the last En stripe to the anterior edge of the telson (which is marked by change in cell density easily seen with Hoechst staining). Cell counts (numbers of nuclei) along this line were also recorded.
3. Growth zone width “A” (GZ width A/cells): this measure is from one lateral edge to another just posterior of the final En stripe. The number of cells in this measure was also recorded. We refer to this measure as the length of the newly formed En stripe.
4. Growth zone width “B” (GZ width B/cells): this measure extends from the one lateral edge of the posterior growth zone to the other, along the boundary of the growth zone and telson. The number of cells in this measure was also recorded.
5. Trunk area: this is a measure of the total ventral area of the larval trunk. The measurement includes the lateral edges of all segments and follows the growth zone width B measurement at the posterior. The final portion of the measure is along the second maxillary En stripe, but not inclusive of that stripe. It measures just posterior to the second maxillary En stripe, but includes the entire ventral area of the first segment.
6. Last segment area (last seg area): this is a measure of the total area of the last segment formed at any specific stage. It is a roughly rectangular measure bounded by the two lateral margins of the segment, growth zone width A and a line just posterior to the penultimate En stripe.
7. Growth zone area (GZ area): this is a roughly trapezoidal measure formed by the two lateral margins of the growth zone and growth zone widths A&B.
8. Last segment length (last segment length/cells): this is a measurement along the midline of the distance between but not including the final two En stripes. The number of cells in this measure was also recorded.

Number of mitotic cells in growth zone: this is a measurement of the number of cells in the ventral epidermis posterior to the last En stripe undergoing mitosis as visualized by Hoechst 33342 (ThermoFisher) or pH3 staining. Note that all mitotic cells were scored at the microscope, focusing down from most ventral to most lateral growth zone tissue.

Length and width measures made by cell counts were used to calculate an estimate for the area of the growth zone in cell numbers (using the formula GZ length × ((GZ width A + GZ width B)/2)) as well as cell field area of the last added segment (last segment length × GZ width A). These were used to estimate the number of cell divisions necessary to add all new segments from the initial GZ cell field.

### Statistics

All scatter plots with lines represent linear regressions of the data; all multiple comparisons are done by analysis of variance and show averages with standard deviation. Statistical analyses were performed using GraphPad Prism 7 software or custom R (3.4.0) code. PCA was conducted with a custom script in R using the ‘prcomp’ function and visualized using the ‘ggbiplot’ package [68]. PCA utilized 8 different morphometric measurements (all measures excluding cell counts and Engrailed number as outlined in *Growth Zone Dimensions* but also excluding number of mitotic cells like pH3, etc.) from 423 individuals that were standardized and compared by axial position (tagma). Axial positions were split into four groups for statistical analysis, an individual “tagma designation” was defined by the position along the body axis of the last added En stripe: En stripes 3–6 = thoracic pre-molt; 7–11 = thoracic post-molt; 12–13 = genital; 14–17 = abdominal.

The following R packages were utilized during data analysis, exploratory data analysis, and visualization; ‘graphics’, ‘devtools’, ‘gridExtra’, ‘data.table’, ‘Hmisc’, ‘extrafont’, ‘broom’, ‘ggplot2’, ‘ggsignif’, and ‘cowplot’. All custom R codes and data are available at https://github.com/savvasjconstantinou/tRinityanalysis.

## Supplementary information

**Additional file 1.** *Thamnocephalus* adds segments linearly. Segment number is plotted against time at one hour intervals and fit with a linear regression. Points are offset to demonstrate the high number of similar measures [72]; n = 20–30 individuals for each time point. Dotted line represents the first molting event at 4 hours. Solid lines represent the transition between tagma, thoracic to genital (~ 12 H) and genital to abdominal (~ 15 H). These data extend the linear rate shown in [37]. Those data were taken under less strictly controlled conditions.

**Additional file 2.** Change in setal morphology that occurs during first molt; used to score animals pre- and post-molt when not tracked as individuals. A, B. Premolt larva showing the relatively smooth trunk (dashed line) and the non-setulated coxal masticatory spine (arrowhead) and basipodial feeding seta (asterisk). C, D. Post-molt larva showing overt trunk morphogenesis in the anterior segments (dashed line) and the setulation of the coxal masticatory spine (arrowhead) and basipodial feeding seta (asterisk). Scale bars = 100 um. E. Average (3.7 h) and standard deviation of time to first molt for a cohort of 46 hatchlings.

**Additional file 3.** Data in manuscript Fig. 3 plotted against time (h post-hatching) instead of developmental stage, as individual points with mean and standard error.

**Additional file 4.** Growth zone length in *Artemia* does not decrease as segments are added. Direct measures of growth zone length in a series of larval stages show that, unlike *Thamnocephalus*, growth zone length is maintained during early segmentation.

**Additional file 5.** Tagma level differences in *Thamnocephalus* morphometric measurements. Tagma level differences (including pre- and post-molt thoracic ‘tagma’ identified from PCA; see Fig. 4) are shown for body length (A), growth zone length (B) and area (C), the width of the newly added En stripe (D), last segment length (E) and area (F). All comparisons are significantly different (Tukey’s HSD; *p* < 0.05) unless otherwise notated with “NS”. The y-axes are measured in mm. Thor Pre = thoracic pre-molt; Thor Post = thoracic post-molt.

**Additional file 6.** PCA biplot grouping by axial position. 423 individuals are plotted along PC1 and PC2 and grouped (in which the measures were made). PC1 explains 64% of the total variance in the data and separates individuals by axial position (segment number); a linear regression of PC1 on segment number indicates that “axial position” is a good predictor of PC1 (adj *R*^2^ = 0.95; *p* < 0.001). Each tagma group is significantly different from one another (Type II MANOVA; *F*_42,1239_ = 38.326, *p* < 0.001).

**Additional file 7.** Correlation of pH3 and Hoechst mitosis counts and cell cycle expression. A. pH3 and Hoechst count correlation for 6 EN animals. We find low correlation at all developmental stages. B. Expression of growth zone pH3 and Hoechst in relation to cell cycle progression. Although pH3 is reported to be expressed throughout M-phase ([37, 73]; red line), we find *Thamnocephalus* pH3 to be expressed early in M-phase (red dotted line). By comparison, mitosis counts using Hoechst only score cells in late M-phase.

**Additional file 8.** Correlation between Hoechst and pH3 mitosis counts within the same individual. For all developmental stages that have both Hoechst and pH3 data, the linear correlation and number of specimens is given.

**Additional file 9.** Estimate of number of times cells in the growth zone of the hatchling would need to divide to produce all the new segmental tissue. Area of the growth zone of the hatchling is assumed to be a trapezoid and the length of the growth zone measured in cells is multiplied by half the sum of the anterior and posterior width of the growth zone, to reach an estimate of 325 cells. Then, length and width in cell diameters of each newly added segment is used to calculate the area of the new segment (as a rectangle). These are summed over all stages measured and the resulting number used to calculate how many times *on average* the cells of the initial growth zone would need to divide to produce all the new tissue.

**Additional file 10.** Three and four hour *Thamnocephalus* larvae double labeled with Edu and anti-Engrailed. Red arrowhead last En stripe; green cells EdU incorporation; yellow line anterior growth zone; blue line posterior growth zone.

**Additional file 11.** Seen without the EdU double labeling, both Wnt4 and WntA show graded expression in the posterior growth zone in *Thamnocephalus*. Expression is quantified using the intensity profile measure in FIJI.

**Additional file 12.** Comparison of dorsal and ventral cell dynamics in *Thamnocephalus* larvae, visualized by EdU incorporation. The pattern of Edu and all growth zone measures carry around to the dorsal side of the larvae (shown in focus in A). Focusing through the same specimen shows the normal pattern we describe in the text (B, cells out of focus due to being viewed through dorsal tissue). This corresponding patterning justifies restricting our measures and calculations to the ventral surface since we focus on changes in dimension and other relative features, not absolute measures.

**Additional file 13.** Confocal image of *Thamnocephalus* larva showing the ectodermal projection is a single continuous epithelial layer (E, outside ellipse) underlaid by a mesodermal layer (M, middle ellipse) and the gut (G, interior ellipse).

**Additional file 14.** Icons of *Thamnocephalus* trunk region with Engrailed staining illustrating the exact position of measures taken to quantify changes in growth zone dimensions (in blue) corresponding to the measures mapped onto an actual photo.

**Additional file 15.** Top table shows number of larvae scored for each timepoint, with age measured as hours post-hatching. The data were collected by carefully staged timepoints. The bottom table shows those same data subsequently binned according to their developmental age, as indicated by counting the number of Engrailed stripes on the trunk.

## Data Availability

All data generated or analyzed during this study are included in this published article [and its Additional files].

## References

[1] Lawrence PA (1992). The making of a fly: the genetics of animal design.

[2] Chipman AD, Fusco G (2008). Thoughts and speculations on the ancestral arthropod segmentation pathway. Evolving pathways: key themes in evolutionary developmental biology.

[3] Scholtz G (1993). Teloblasts in decapod embryos: an embryonic character reveals the monophyletic origin of freshwater crayishes (Crustacea, Decapoda). Zool Anz.

[4] Snodgrass RE (1938). Evolution of the annelida, onychophora, and arthropoda. Smithson Misc Collect.

[5] Anderson DT (1967). Larval development and segment formation in the branchiopod crustaceans *Limnadia stanleyana* King (Conchostraca) and Artemia salina (L.) (Anostraca). Aust. J Zool.

[6] Anderson DT (1973). Embryology and phylogeny in annelids and arthropods.

[7] Davis GK, Patel NH (2002). Short, long, and beyond: molecular and embryological approaches to insect segmentation. Annu Rev Entomol.

[8] Liu PZ, Kaufman TC (2005). Short and long germ segmentation: unanswered questions in the evolution of a developmental mode. Evol Dev.

[9] Nagy LM, Kiguchi K, Riddiford L (1994). Morphogenesis in the early embryo of the lepidopteran Bombyx mori. Dev Biol.

[10] Tautz D, Friedrich M, Schröder R (1994). Insect embryogenesis—what is ancestral and what is derived?. Development.

[11] Sarrazin AF, Peel AD, Averof M (2012). A segmentation clock with two-segment periodicity in insects. Science.

[12] Nakamoto A, Hester SD, Constantinou SJ, Blaine WG, Tewksbury AB, Matei MT, Nagy LM, Williams TA (2015). Changing cell behaviors during beetle embryogenesis correlates with slowing of segmentation. Nat Commun.

[13] Benton MA (2018). A revised understanding of *Tribolium* morphogenesis further reconciles short and long germ development. PLoS Biol.

[14] Peel AD, Chipman AD, Akam M (2005). Arthropod segmentation: beyond the Drosophila paradigm. Nat Rev Genet.

[15] McGregor AP, Hilbrant M, Pechmann M, Schwager EE, Prpic NM, Damen WGM (2008). *Cupiennius salei* and *Achaearanea tepidariorum*: spider models for investigating evolution and development. BioEssays.

[16] Janssen R, Le Gouar M, Pechmann M, Poulin F, Bolognesi R, Schwager E, Hopfen C, Colbourne J, Budd G, Brown S (2010). Conservation, loss, and redeployment of Wnt ligands in protostomes: implications for understanding the evolution of segment formation. BMC Evol Biol.

[17] Chipman AD, Wanninger A (2015). Hexapoda: comparative aspects of early development. Evolutionary developmental biology of invertebrates.

[18] Damen WG (2007). Evolutionary conservation and divergence of the segmentation process in arthropods. Dev Dyn.

[19] Williams TA, Nagy LM (2017). Linking gene regulation to cell behaviors in the posterior growth zone of sequentially segmenting arthropods. Arthropod Struct Dev.

[20] Auman T, Chipman AD (2017). The evolution of gene regulatory networks that deine arthropod body plans. Integr Comp Biol.

[21] Clark E, Peel AD, Akam M (2019). Arthropod segmentation. Development.

[22] McGregor AP, Pechmann M, Schwager EE, Damen WGM (2009). An ancestral regulatory network for posterior development in arthropods. Commun Integr Biol.

[23] Shinmyo Y, Mito T, Matsushita T, Sarashina I, Miyawaki K, Ohuchi H, Noji S (2005). *caudal* is required for gnathal and thoracic patterning and for posterior elongation in the intermediate-germband cricket *Gryllus bimaculatus*. Mech Dev.

[24] Chesebro JE, Pueyo JI, Couso JP (2013). Interplay between a Wnt-dependent organiser and the Notch segmentation clock regulates posterior development in Periplaneta americana. Biol Open.

[25] Auman T, Vreede BMI, Weiss A, Hester SD, Williams TA, Nagy LM, Chipman AD (2017). Dynamics of growth zone patterning in the milkweed bug Oncopeltus fasciatus. Development.

[26] Giribet G, Edgecombe GD (2019). The phylogeny and evolutionary history of arthropods. Curr Biol.

[27] Lozano-Fernandez J, Giacomelli M, Fleming JF, Chen A, Vinther J, Thomsen PF, Glenner H, Palero F, Legg DA, Ilife TM, Pisani D, Olesen J (2019). Pancrustacean evolution illuminated by taxon-rich genomic-scale data sets with an expanded remipede sampling. Genome Biol Evol.

[28] Rogers DC, Likens GF (2009). Branchiopoda (Anostraca, Notostraca, Laevicaudata, Spinicaudata, and Cyclestherida). Encyclopedia of inland waters.

[29] Alvarenga P, Mourinha C, Farto M, Palma P, Sengo J, Marie-Christine MC, Cunha-Queda C (2016). Ecotoxicological assessment of the potential impact on soil porewater, surface and groundwater from the use of organic wastes as soil amendments. Ecotoxicol Environ Saf.

[30] Linder F (1941). Contributions to the morphology and the taxonomy of the Branchiopoda Anostraca. Zool Bidrag Fran Uppsala..

[31] Fryer G (1983). Functional ontogenetic changes in *Branchinecta ferox* (Milne-Edwards) (Crustacea: Anostraca). Phil Trans R Soc Lond B.

[32] Møller OS, Olesen J, Høeg JT (2004). On the larval development of *Eubranchipus grubii* (Crustacea, Branchiopoda, Anostraca), with notes on the basal phylogeny of the Branchiopoda. Zoomorphology..

[33] Freeman JA (1986). Epidermal cell proliferation during thoracic development in larvae of Artemia. J Crust Biol.

[34] Williams TA (2007). Limb morphogenesis in the branchiopod crustacean, Thamnocephalus platyurus, and the evolution of proximal limb lobes within Anostraca. J Zool Syst Evol Res.

[35] Constantinou SJ, Williams TA, Pace RM, Stangl AJ, Nagy LM (2016). Wnt repertoire and developmental expression patterns in the crustacean Thamnocephalus platyurus. Evol Dev.

[36] Freeman JA, Cheshire LB, Macrae TH (1992). Epithelial morphogenesis in developing Artemia—the role of cell replication, cell-shape change, and the cytoskeleton. Dev Biol.

[37] Williams TA, Blachuta B, Hegna TA, Nagy LM (2012). Decoupling elongation and segmentation: Notch involvement in anostracan crustacean segmentation. Evol Dev.

[38] Hendzel MJ, Wei Y, Mancini MA, Van Hooser A, Ranalli T, Brinkley BR, Bazett-Jones DP, Allis CD (1997). Mitosis-specific phosphorylation of histone H3 initiates primarily within pericentromeric heterochromatin during G2 and spreads in an ordered fashion coincident with mitotic chromosome condensation. Chromosoma.

[39] Le LT, Vu HL, Nguyen CH, Molla A (2013). Basal aurora kinase B activity is suicient for histone H3 phosphorylation in prophase. Biol Open.

[40] Hans F, Dimitrov S (2001). Histone H3 phosphorylation and cell division. Oncogene.

[41] Irvine KD, Wieschaus E (1994). Cell intercalation during Drosophila germband extension and its regulation by pair-rule segmentation genes. Development.

[42] Benton MA, Akam M, Pavlopoulos A (2013). Cell and tissue dynamics during Tribolium embryogenesis revealed by versatile luorescence labeling approaches. Development.

[43] Benton MA, Pechmann M, Frey N, Stappert D, Conrads KH, Chen Y, Stamataki E, Pavlopoulos A, Roth S (2016). Toll genes have an ancestral role in axis elongation. Curr Biol.

[44] Cepeda RE, Pardo RV, Macaya CC, Sarrazin AF (2017). Contribution of cell proliferation to axial elongation in the red flour beetle *Tribolium castaneum*. PLoS ONE.

[45] Mayer G, Kato C, Quast B (2010). Growth patterns in Onychophora (velvet worms): lack of a localised posterior proliferation zone. BMC Evol Biol.

[46] Rosenberg MI, Brent AE, Payre F, Desplan C (2014). Dual mode of embryonic development is highlighted by expression and function of Nasonia pairrule genes. Elife.

[47] Bouldin CM, Snelson CD, Farr GH, Kimelman D (2014). Restricted expression of cdc25a in the tailbud is essential for formation of the zebraish posterior body. Genes Dev.

[48] Foe VE (1989). Mitotic domains reveal early commitment of cells in Drosophila embryos. Development.

[49] Foe VE, Odell G (1989). Mitotic domains partition fly embryos, reflecting early cell biological consequences of determination in progress. Am Zool.

[50] Sommer R, Tautz D (1991). Asynchronous mitotic domains during blastoderm formation in Musca domestica L. (Diptera). Roux Arch Dev Biol.

[51] Hayden L, Schlosser G, Arthur W (2015). Functional analysis of centipede development supports roles for Wnt genes in posterior development and segment generation. Evol Dev.

[52] Hayden L, Arthur W (2014). The centipede Strigamia maritima possesses a large complement of Wnt genes with diverse expression patterns. Evol Dev.

[53] Janssen R, Prpic NM, Damen WGM (2004). Gene expression suggests decoupled dorsal and ventral segmentation in the millipede Glomeris marginata (Myriapoda: Diplopoda). Dev Biol.

[54] Janssen R, Posnien N (2014). Identiication and embryonic expression of Wnt2, Wnt4, Wnt5 and Wnt9 in the millipede Glomeris marginata (Myriapoda: Diplopoda). Gene Expr Patterns.

[55] Bolognesi R, Farzana L, Fischer TD, Brown SJ (2008). Multiple Wnt genes are required for segmentation in the short-germ embryo of Tribolium castaneum. Curr Biol.

[56] Stollewerk A, Schoppmeier M, Damen WG (2003). Involvement of Notch and Delta genes in spider segmentation. Nature.

[57] Copf T, Schröder R, Averof M (2004). Ancestral role of caudal genes in axis elongation and segmentation. Proc Natl Acad Sci USA.

[58] Olesnicky EC, Brent AE, Tonnes L, Walker M, Pultz MA, Leaf D, Desplan C (2006). A caudal mRNA gradient controls posterior development in the wasp Nasonia. Development.

[59] Schoppmeier M, Fischer S, Schmitt-Engel C, Löhr U, Klingler M (2009). An ancient anterior patterning system promotes caudal repression and head formation in ecdysozoa. Curr Biol.

[60] da Silva SM, Vincent JP (2007). Oriented cell divisions in the extending germband of Drosophila. Development.

[61] Dohle W, Gerberding M, Hejnol Scholtz G, Scholtz G (2004). Cell lineage, segment differentiation, and gene expression in crustaceans. Evolutionary developmental biology of crustacea.

[62] Scholtz G, Dohle W (1996). Cell lineage and cell fate in crustacean embryos—a comparative approach. Int J Dev Biol.

[63] Wilson V, Olivera-Martinez I, Storey KG (2009). Stem cells, signals and vertebrate body axis extension. Development.

[64] Jurberg AD, Aires R, Varela-Lasheras I, Novoa A, Mallo M (2013). Switching axial progenitors from producing trunk to tail tissues in vertebrate embryos. Dev Cell.

[65] McPherron AC, Lawler AM, Lee SJ (1999). Regulation of anterior/posterior patterning of the axial skeleton by growth/diferentiation factor 11. Nat Genet.

[66] Weisz P (1946). The space-time pattern of segment formation in Artemia salina. Biol Bull.

[67] Brena C, Akam M (2013). An analysis of segmentation dynamics throughout embryogenesis in the centipede Strigamia maritima. BMC Biol.

[68] Weisblat DA, Kuo DH (2014). Developmental biology of the leech Helobdella. Int J Dev Biol.

[69] Özpolat BD, Handberg-Thorsager M, Vervoort M, Balavoine G (2017). Cell lineage and cell cycling analyses of the 4d micromere using live imaging in the marine annelid Platynereis dumerilii. Elife.

[70] Williams T, Nulsen C, Nagy LM (2002). A complex role for distal-less in crustacean appendage development. Dev Biol.

[71] Dahms HU, Fornshell JA, Fornshell BJ (2006). Key for the identiication of crustacean nauplii. Organ Divers Evol.

[72] Winston C. Extrafont: tools for using fonts. R package version 0.17. 2014. https://CRAN.R-project.org/package=extrafont.

[73] Giet R, Glover DM (2001). Drosophila aurora B kinase is required for histone H3 phosphorylation and condensin recruitment during chromosome condensation and to organize the central spindle during cytokinesis. J Cell Biol.

