# Supplementary material for: Elongation during segmentation shows axial variability, low mitotic rates, and synchronized cell cycle domains in the crustacean, Thamnocephalus platyurus
Source: EvoDevo. 2020 Jan 18;11:1. doi: 10.1186/s13227-020-0147-0 (PMC6969478; doi:10.1186/s13227-020-0147-0)

**Additional files:**

**Additional file 1**. ***Thamnocephalus* adds segments linearly.** Segment number is plotted against time at one hour intervals and fit with a linear regression. Points are offset to demonstrate the high number of similar measures (Winston, 2014); n=20-30 individuals for each time point. Dotted line represents the first molting event at 4 hours. Solid lines represent the transition between tagma, thoracic to genital (~12H) and genital to abdominal (~15H). These data extend the linear rate shown in Williams et al., 2012. Those data were taken under less strictly controlled conditions.

References:

Williams TA, Blachuta B, Hegna TA, Nagy, LM. Decoupling elongation and segmentation: *Notch* involvement in anostracan crustacean segmentation*. Evol. Dev.* 2012;*14*:372-382.

**Winston, C.**  extrafont: Tools for using fonts. *R package* version 0.17. Available at 2014: https://CRAN.R-project.org/package=extrafont


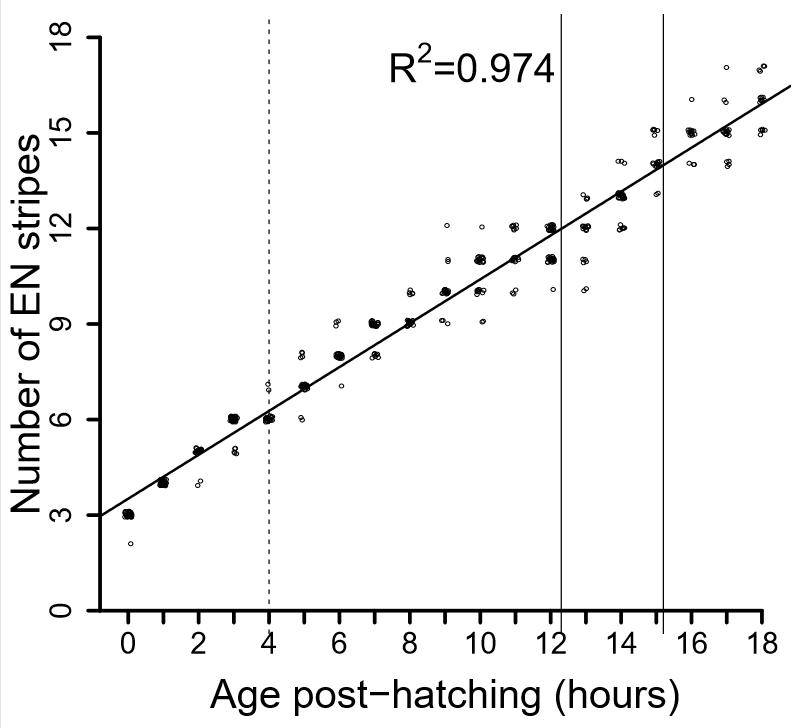

Supplement: Supplementary file 1 — Additional file 1. Thamnocephalus adds segments linearly. Segment number is plotted against time at one hour intervals and fit with a linear regression. Points are offset to demonstrate the high number of similar measures [72]; n = 20–30 individuals for each time point. Dotted line represents the first molting event at 4 hours. Solid lines represent the transition between tagma, thoracic to genital (~ 12 H) and genital to abdominal (~ 15 H). These data extend the linear rate shown in [37]. Those data were taken under less strictly controlled conditions. [file 13227_2020_147_MOESM1_ESM.docx]
