# Supplementary material for: Elongation during segmentation shows axial variability, low mitotic rates, and synchronized cell cycle domains in the crustacean, Thamnocephalus platyurus
Source: EvoDevo. 2020 Jan 18;11:1. doi: 10.1186/s13227-020-0147-0 (PMC6969478; doi:10.1186/s13227-020-0147-0)

**Additional file 2**. **Change in setal morphology that occurs during first molt; used to score animals pre- and post-molt when not tracked as individuals.** A, B. Premolt larva showing the relatively smooth trunk (dashed line) and the non-setulated coxal masticatory spine (arrowhead) and basipodial feeding seta (asterisk). C, D. Post-molt larva showing overt trunk morphogenesis in the anterior segments (dashed line) and the setulation of the coxal masticatory spine (arrowhead) and basipodial feeding seta (asterisk). Scale bars = 100 um. E. Average (3.7h) and standard deviation of time to first molt for a cohort of 46 hatchlings.


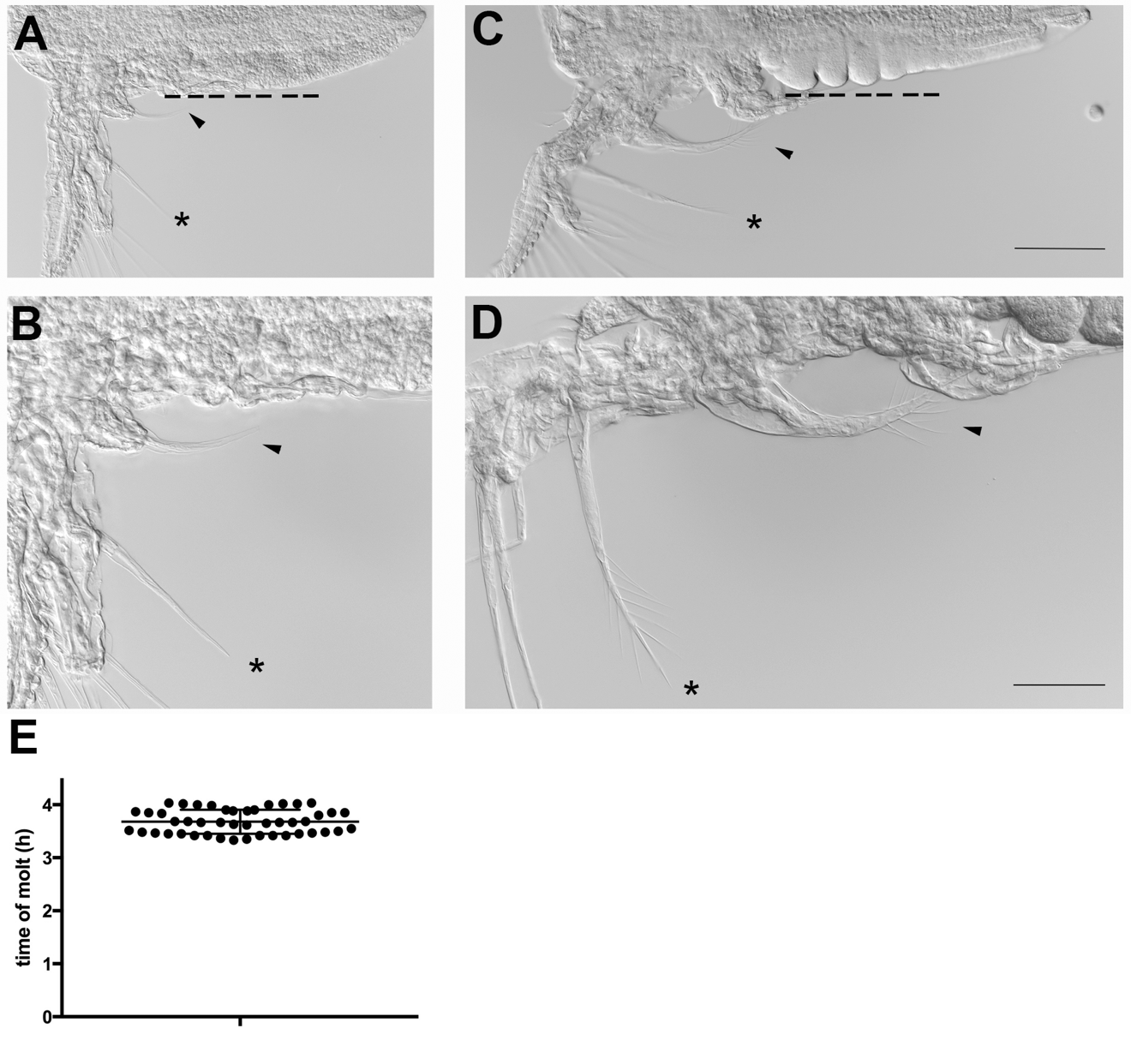

Supplement: Supplementary file 2 — Additional file 2. Change in setal morphology that occurs during first molt; used to score animals pre- and post-molt when not tracked as individuals. A, B. Premolt larva showing the relatively smooth trunk (dashed line) and the non-setulated coxal masticatory spine (arrowhead) and basipodial feeding seta (asterisk). C, D. Post-molt larva showing overt trunk morphogenesis in the anterior segments (dashed line) and the setulation of the coxal masticatory spine (arrowhead) and basipodial feeding seta (asterisk). Scale bars = 100 um. E. Average (3.7 h) and standard deviation of time to first molt for a cohort of 46 hatchlings. [file 13227_2020_147_MOESM2_ESM.docx]
