# Supplementary material for: Elongation during segmentation shows axial variability, low mitotic rates, and synchronized cell cycle domains in the crustacean, Thamnocephalus platyurus
Source: EvoDevo. 2020 Jan 18;11:1. doi: 10.1186/s13227-020-0147-0 (PMC6969478; doi:10.1186/s13227-020-0147-0)

**Additional file 3**. Data in manuscript Fig. 3 plotted against time (h post-hatching) instead of developmental stage as in the manuscript, as individual points with mean and standard error.


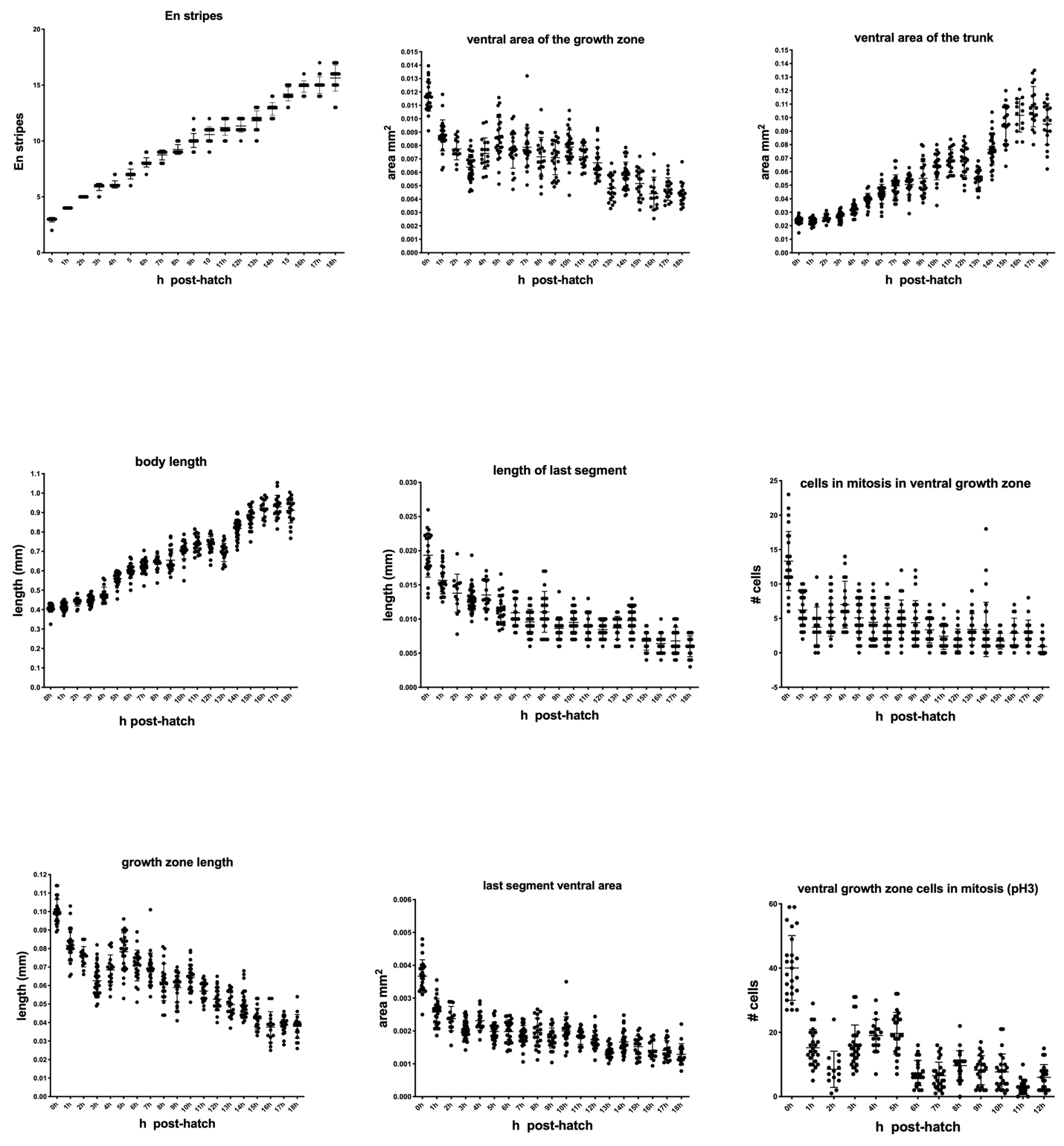

Supplement: Supplementary file 3 — Additional file 3. Data in manuscript Fig. 3 plotted against time (h post-hatching) instead of developmental stage, as individual points with mean and standard error. [file 13227_2020_147_MOESM3_ESM.docx]
