# Supplementary material for: Elongation during segmentation shows axial variability, low mitotic rates, and synchronized cell cycle domains in the crustacean, Thamnocephalus platyurus
Source: EvoDevo. 2020 Jan 18;11:1. doi: 10.1186/s13227-020-0147-0 (PMC6969478; doi:10.1186/s13227-020-0147-0)

**Additional file 4**. **Growth zone length in *Artemia* does not decrease as segments are added.** Direct measures of growth zone length in a series of larval stages show that, unlike *Thamnocephalus*, growth zone length is maintained during early segmentation.


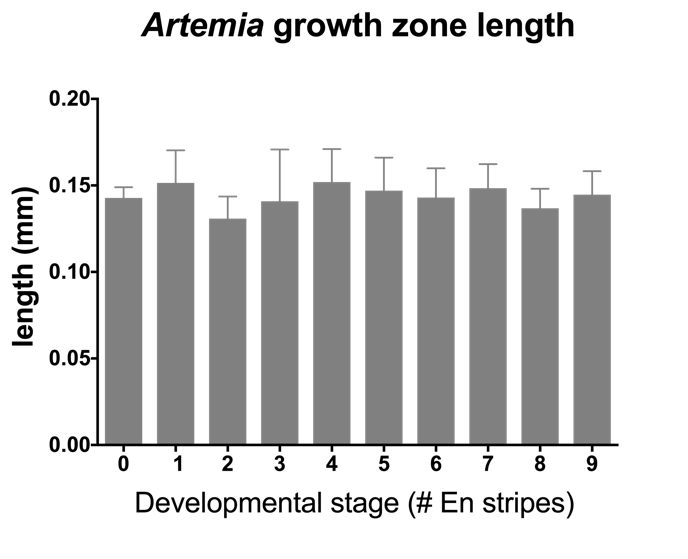

Supplement: Supplementary file 4 — Additional file 4. Growth zone length in Artemia does not decrease as segments are added. Direct measures of growth zone length in a series of larval stages show that, unlike Thamnocephalus, growth zone length is maintained during early segmentation. [file 13227_2020_147_MOESM4_ESM.docx]
