# Supplementary material for: Elongation during segmentation shows axial variability, low mitotic rates, and synchronized cell cycle domains in the crustacean, Thamnocephalus platyurus
Source: EvoDevo. 2020 Jan 18;11:1. doi: 10.1186/s13227-020-0147-0 (PMC6969478; doi:10.1186/s13227-020-0147-0)

**Additional file 5. Tagma level differences in *Thamnocephalus* morphometric measurements.** Tagma level differences (including pre- and post-molt thoracic ‘tagma’ identified from PCA; see Figure 4) are shown for body length (A), growth zone length (B) and area (C), the width of the newly added En stripe (D), last segment length (E) and area (F). All comparisons are significantly different (Tukey’s HSD; p<0.05) unless otherwise notated with “NS.”. The y-axes are measured in mm. Thor Pre= thoracic pre-molt; Thor Post= thoracic post-molt.


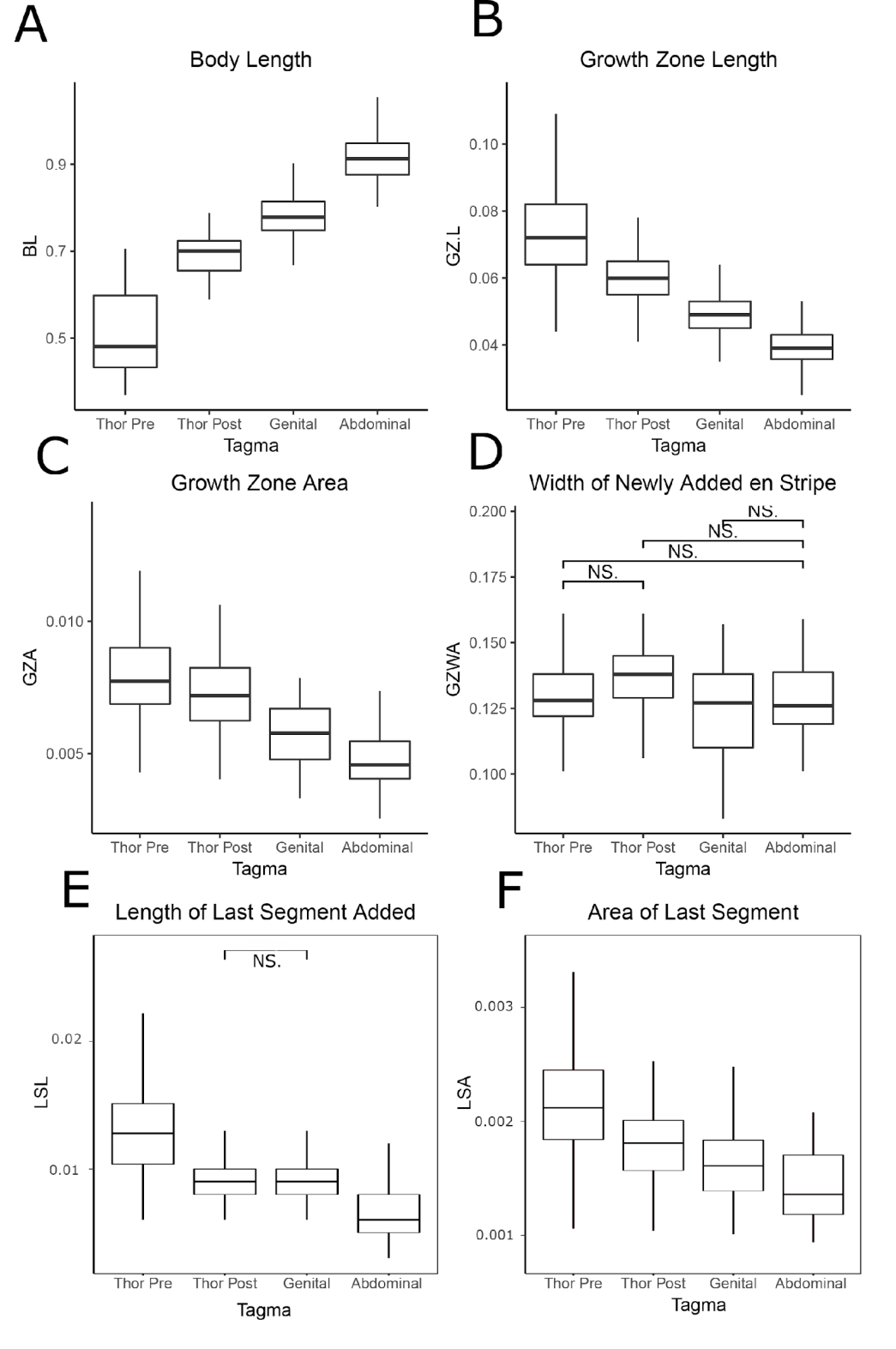

Supplement: Supplementary file 5 — Additional file 5. Tagma level differences in Thamnocephalus morphometric measurements. Tagma level differences (including pre- and post-molt thoracic ‘tagma’ identified from PCA; see Fig. 4) are shown for body length (A), growth zone length (B) and area (C), the width of the newly added En stripe (D), last segment length (E) and area (F). All comparisons are significantly different (Tukey’s HSD; p < 0.05) unless otherwise notated with “NS”. The y-axes are measured in mm. Thor Pre = thoracic pre-molt; Thor Post = thoracic post-molt. [file 13227_2020_147_MOESM5_ESM.docx]
