# Supplementary material for: Elongation during segmentation shows axial variability, low mitotic rates, and synchronized cell cycle domains in the crustacean, Thamnocephalus platyurus
Source: EvoDevo. 2020 Jan 18;11:1. doi: 10.1186/s13227-020-0147-0 (PMC6969478; doi:10.1186/s13227-020-0147-0)
Supplement: Supplementary file 6 — Additional file 6. PCA biplot grouping by axial position. 423 individuals are plotted along PC1 and PC2 and grouped (in which the measures were made). PC1 explains 64% of the total variance in the data and separates individuals by axial position (segment number); a linear regression of PC1 on segment number indicates that “axial position” is a good predictor of PC1 (adj R2 = 0.95; p < 0.001). Each tagma group is significantly different from one another (Type II MANOVA; F42,1239 = 38.326, p < 0.001). [file 13227_2020_147_MOESM6_ESM.docx]

**Additional file 6**. **PCA biplot grouping by axial position.** 423 individuals are plotted along PC1 and PC2 and grouped (in which the measures were made). PC1 explains 64% of the total variance in the data and separates individuals by axial position (segment number); a linear regression of PC1 on segment number indicates that “axial position” is a good predictor of PC1 (adj R^2^= 0.95; p<0.001). Each tagma group is significantly different from one another (Type II MANOVA; F_42,1239_=38.326, p<0.001).

**
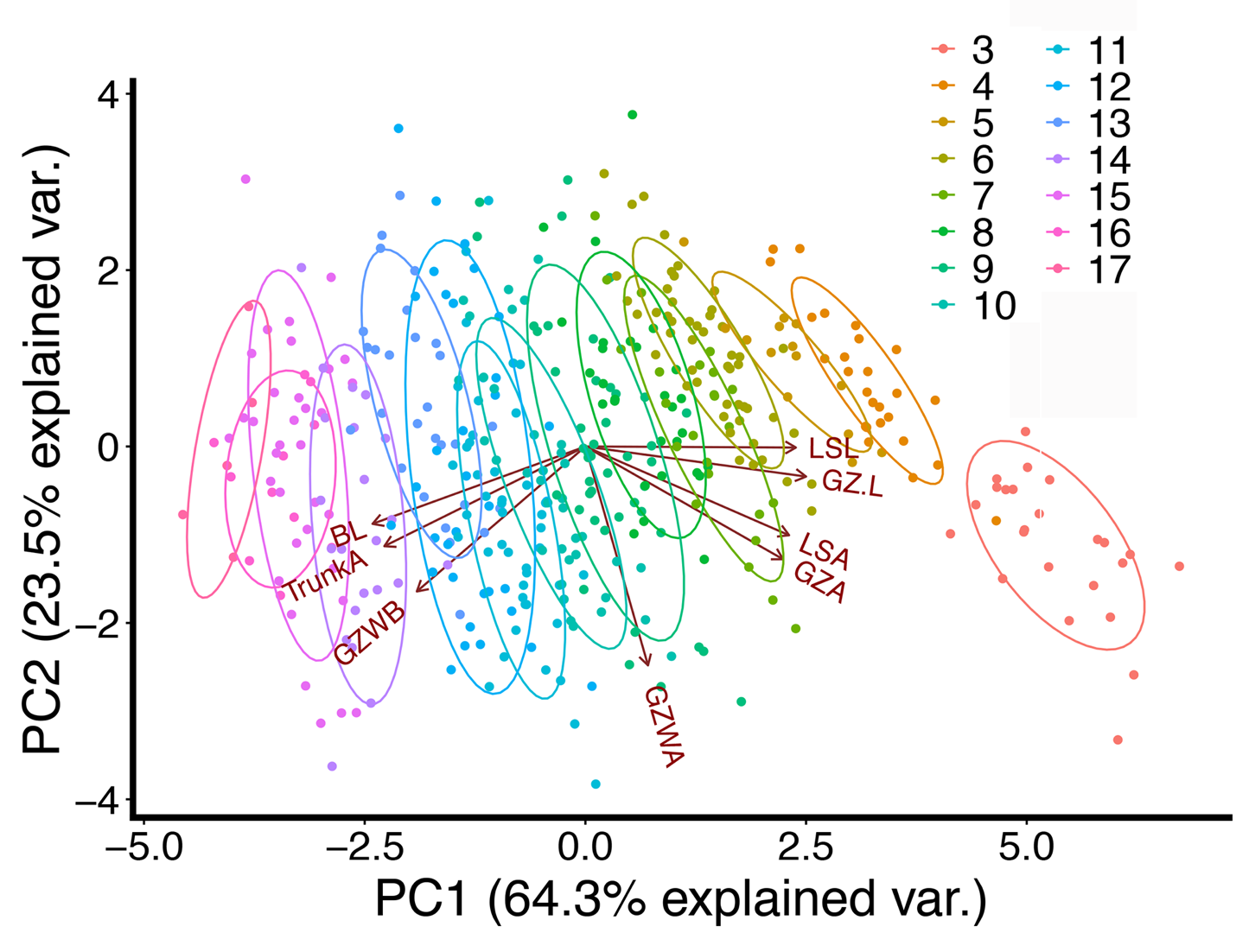
**
