# Supplementary material for: Elongation during segmentation shows axial variability, low mitotic rates, and synchronized cell cycle domains in the crustacean, Thamnocephalus platyurus
Source: EvoDevo. 2020 Jan 18;11:1. doi: 10.1186/s13227-020-0147-0 (PMC6969478; doi:10.1186/s13227-020-0147-0)
Supplement: Supplementary file 7 — Additional file 7. Correlation of pH3 and Hoechst mitosis counts and cell cycle expression. A. pH3 and Hoechst count correlation for 6 EN animals. We find low correlation at all developmental stages. B. Expression of growth zone pH3 and Hoechst in relation to cell cycle progression. Although pH3 is reported to be expressed throughout M-phase ([37, 73]; red line), we find Thamnocephalus pH3 to be expressed early in M-phase (red dotted line). By comparison, mitosis counts using Hoechst only score cells in late M-phase. [file 13227_2020_147_MOESM7_ESM.docx]

**Additional file 7. Correlation of pH3 and Hoechst mitosis counts and cell cycle expression.** A. pH3 and Hoechst count correlation for 6 EN animals. We find low correlation at all developmental stages. B. Expression of growth zone pH3 and Hoechst in relation to cell cycle progression. Although pH3 is reported to be expressed throughout M-phase (Hendzelm et al., 1997; Giet and Glover, 2001; red line), we find *Thamnocephalus* pH3 to be expressed early in M-phase (red dotted line). By comparison, mitosis counts using Hoechst only score cells in late M-phase.


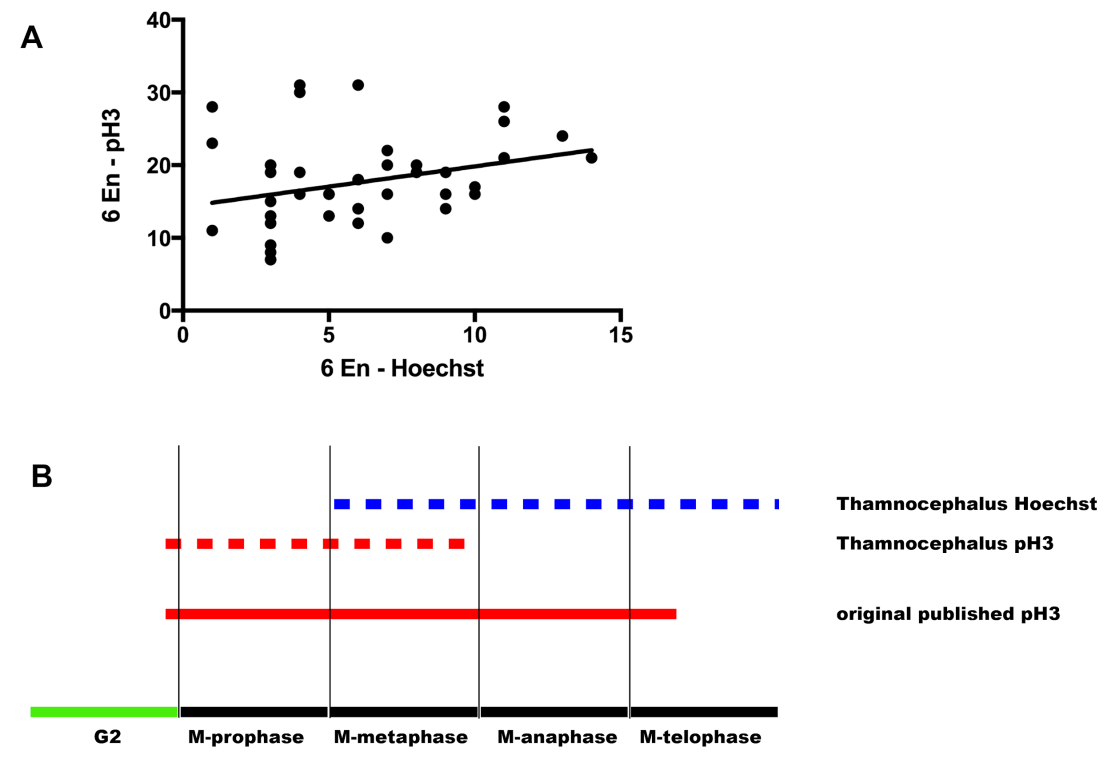


References:

Giet R, Glover DM. 2001. Drosophila aurora B kinase is required for histone H3 phosphorylation and condensin recruitment during chromosome condensation and to organize the central spindle during cytokinesis. J Cell Biol. 2001 Feb 19;152(4):669-82.

M.J. Hendzel, Y. Wei, M.A. Mancini, A. Van Hooser, T. Ranalli, B.R. Brinkley, D.P. Bazett-Jones, C.D. Allis. 1997. Mitosis-specific phosphorylation of histone H3 initiates primarily within pericentromeric heterochromatin during G2 and spreads in an ordered fashion coincident with mitotic chromosome condensation. Chromosoma 106:348–360, pmid:9362543.
