# Supplementary material for: Elongation during segmentation shows axial variability, low mitotic rates, and synchronized cell cycle domains in the crustacean, Thamnocephalus platyurus
Source: EvoDevo. 2020 Jan 18;11:1. doi: 10.1186/s13227-020-0147-0 (PMC6969478; doi:10.1186/s13227-020-0147-0)
Supplement: Supplementary file 8 — Additional file 8. Correlation between Hoechst and pH3 mitosis counts within the same individual. For all developmental stages that have both Hoechst and pH3 data, the linear correlation and number of specimens is given. [file 13227_2020_147_MOESM8_ESM.docx]

**Additional file 8**. **Correlation between Hoechst and pH3 mitosis counts within the same individual.** For all developmental stages that have both Hoechst and pH3 data, the linear correlation and number of specimens is given.

| **Developmental stage** | **R^2^ value for linear correlation between mitosis counts from Hoechst figures versus pH3 staining in the same specimens** | **Number of specimens** |
| --- | --- | --- |
| 3 En | 0.168 | 24 |
| 4 En | 0.215 | 25 |
| 5 En | 0.656 | 14 |
| 6 En | 0.084 | 44 |
| 7 En | 0.340 | 24 |
| 8 En | 0.063 | 29 |
| 9 En | 0.413 | 44 |
| 10 En | 0.322 | 34 |
| 11 En | 0.429 | 39 |
| 12 En | 0.084 | 17 |
