# Supplementary material for: Elongation during segmentation shows axial variability, low mitotic rates, and synchronized cell cycle domains in the crustacean, Thamnocephalus platyurus
Source: EvoDevo. 2020 Jan 18;11:1. doi: 10.1186/s13227-020-0147-0 (PMC6969478; doi:10.1186/s13227-020-0147-0)

**Additional file 9.** Estimate of number of times cells in the growth zone of the hatchling would need to divide to produce all the new segmental tissue. Area of the growth zone of the hatchling is assumed to be a trapezoid and the length of the growth zone measured in cells is multiplied by half the sum of the anterior and posterior width of the growth zone, to reach an estimate of 325 cells. Then, length and width in cell diameters of each newly added segment is used to calculate the area of the new segment (as a rectangle). These are summed over all stages measured and the resulting number used to calculate how many times *on average* the cells of the initial growth zone would need to divide to produce all the new tissue.


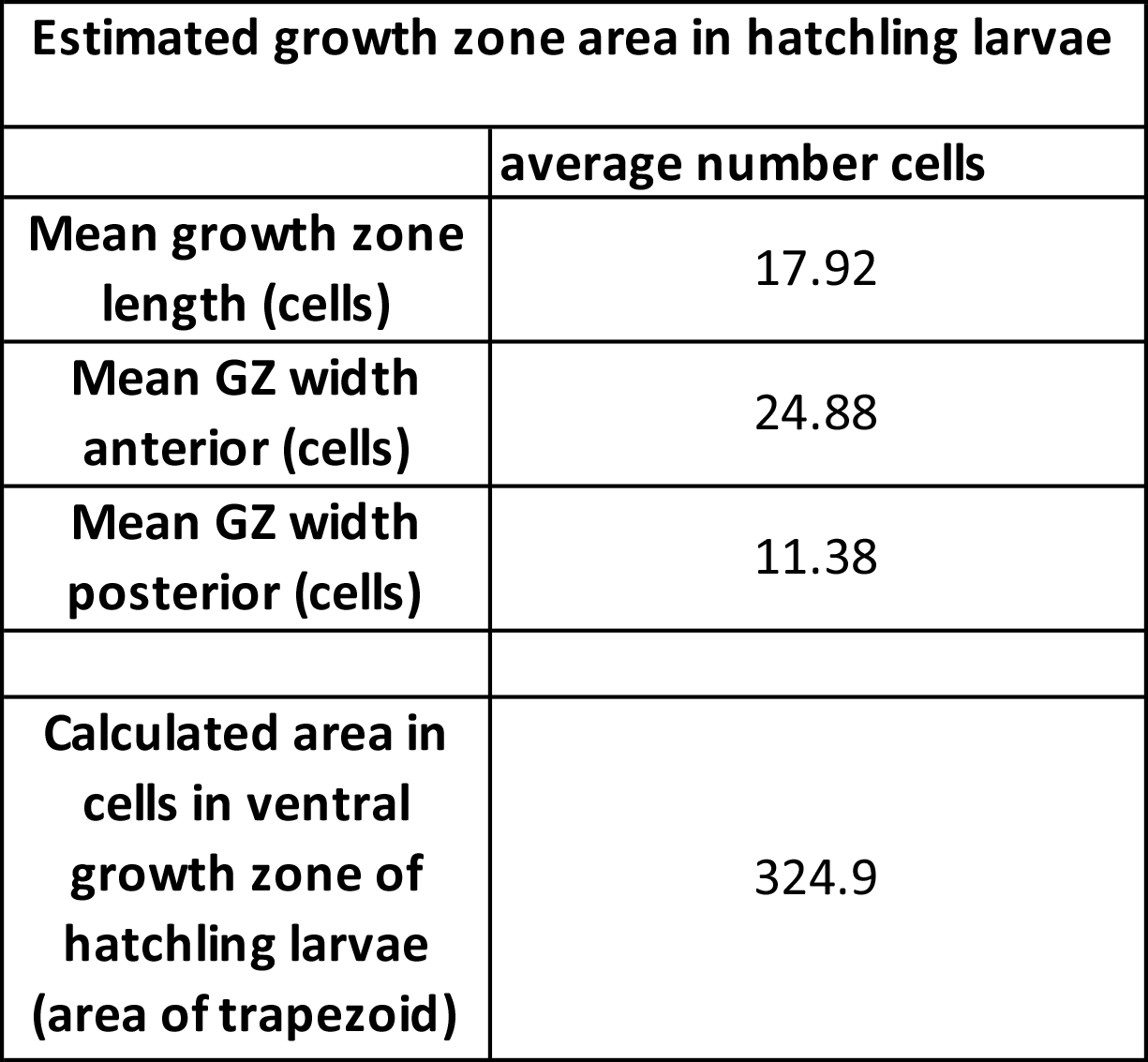


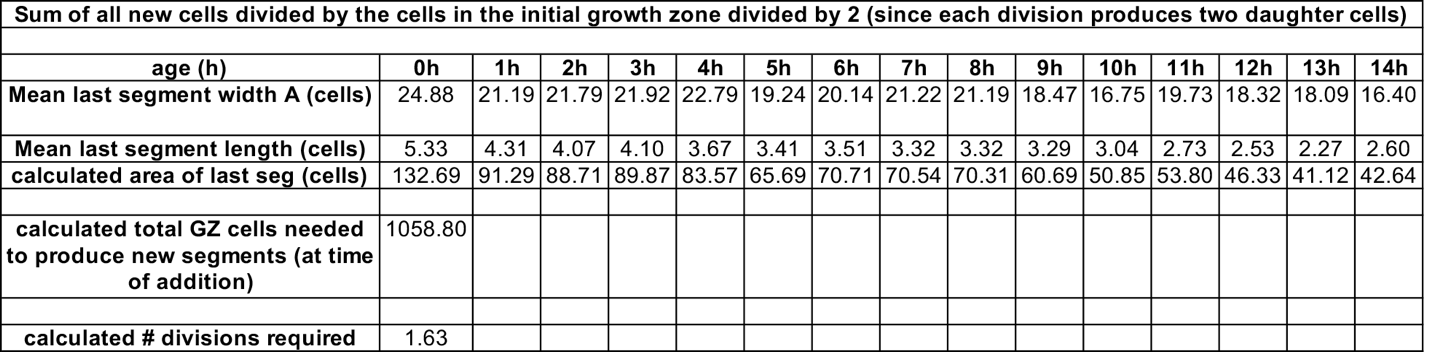

Supplement: Supplementary file 9 — Additional file 9. Estimate of number of times cells in the growth zone of the hatchling would need to divide to produce all the new segmental tissue. Area of the growth zone of the hatchling is assumed to be a trapezoid and the length of the growth zone measured in cells is multiplied by half the sum of the anterior and posterior width of the growth zone, to reach an estimate of 325 cells. Then, length and width in cell diameters of each newly added segment is used to calculate the area of the new segment (as a rectangle). These are summed over all stages measured and the resulting number used to calculate how many times on average the cells of the initial growth zone would need to divide to produce all the new tissue. [file 13227_2020_147_MOESM9_ESM.docx]
