# Supplementary material for: Elongation during segmentation shows axial variability, low mitotic rates, and synchronized cell cycle domains in the crustacean, Thamnocephalus platyurus
Source: EvoDevo. 2020 Jan 18;11:1. doi: 10.1186/s13227-020-0147-0 (PMC6969478; doi:10.1186/s13227-020-0147-0)

**Additional file 10**. Three and four hour Thamnocephalus larvae double labeled with Edu and anti-Engrailed. Red arrowhead­ last En stripe; green cells EdU incorporation; yellow line anterior growth zone; blue line posterior growth zone.


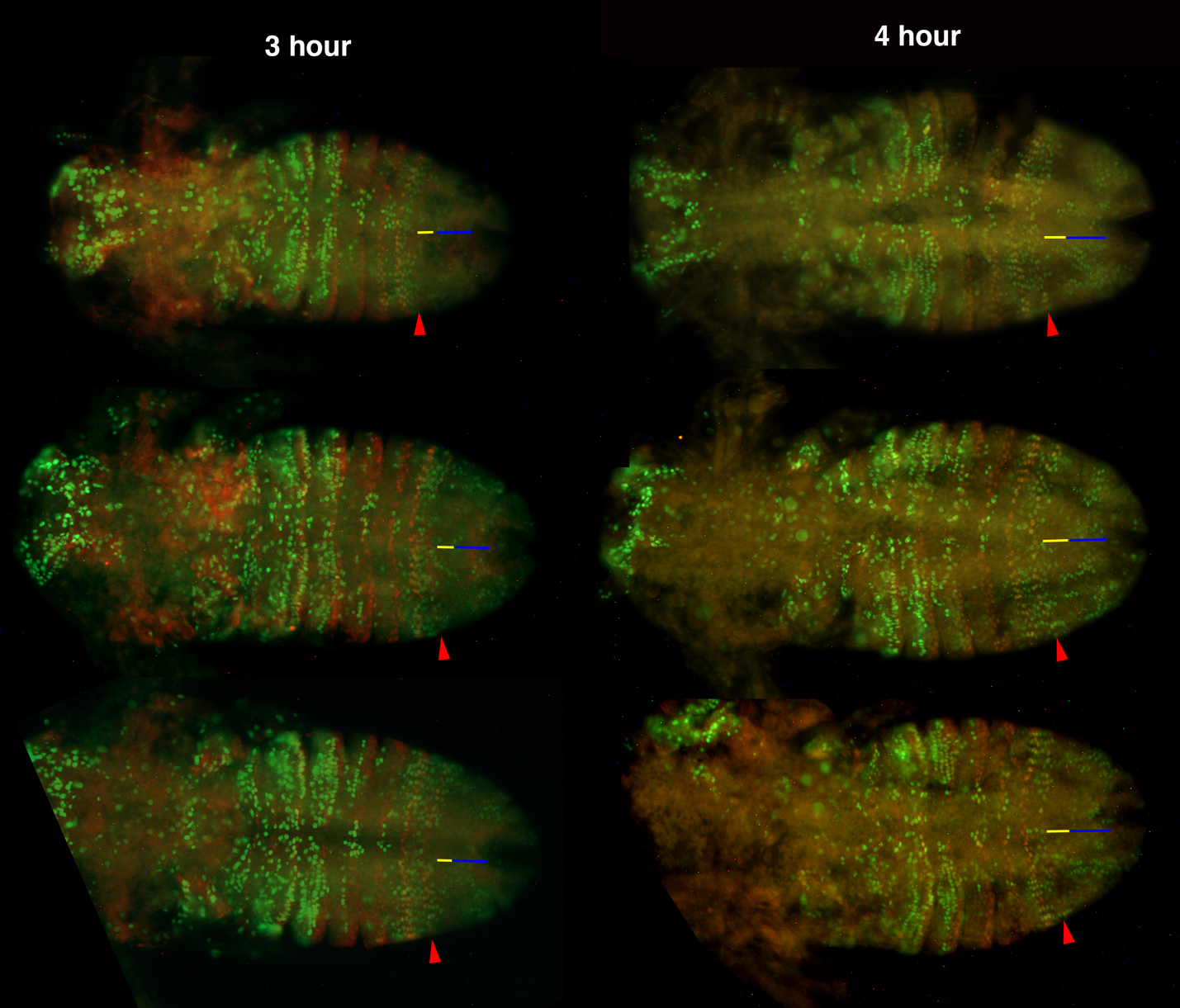

Supplement: Supplementary file 10 — Additional file 10. Three and four hour Thamnocephalus larvae double labeled with Edu and anti-Engrailed. Red arrowhead last En stripe; green cells EdU incorporation; yellow line anterior growth zone; blue line posterior growth zone. [file 13227_2020_147_MOESM10_ESM.docx]
