# Supplementary material for: Elongation during segmentation shows axial variability, low mitotic rates, and synchronized cell cycle domains in the crustacean, Thamnocephalus platyurus
Source: EvoDevo. 2020 Jan 18;11:1. doi: 10.1186/s13227-020-0147-0 (PMC6969478; doi:10.1186/s13227-020-0147-0)

**Additional file 11.** Seen without the EdU double labeling, both Wnt4 and WntA show graded expression in the posterior growth zone in *Thamnocephalus*.


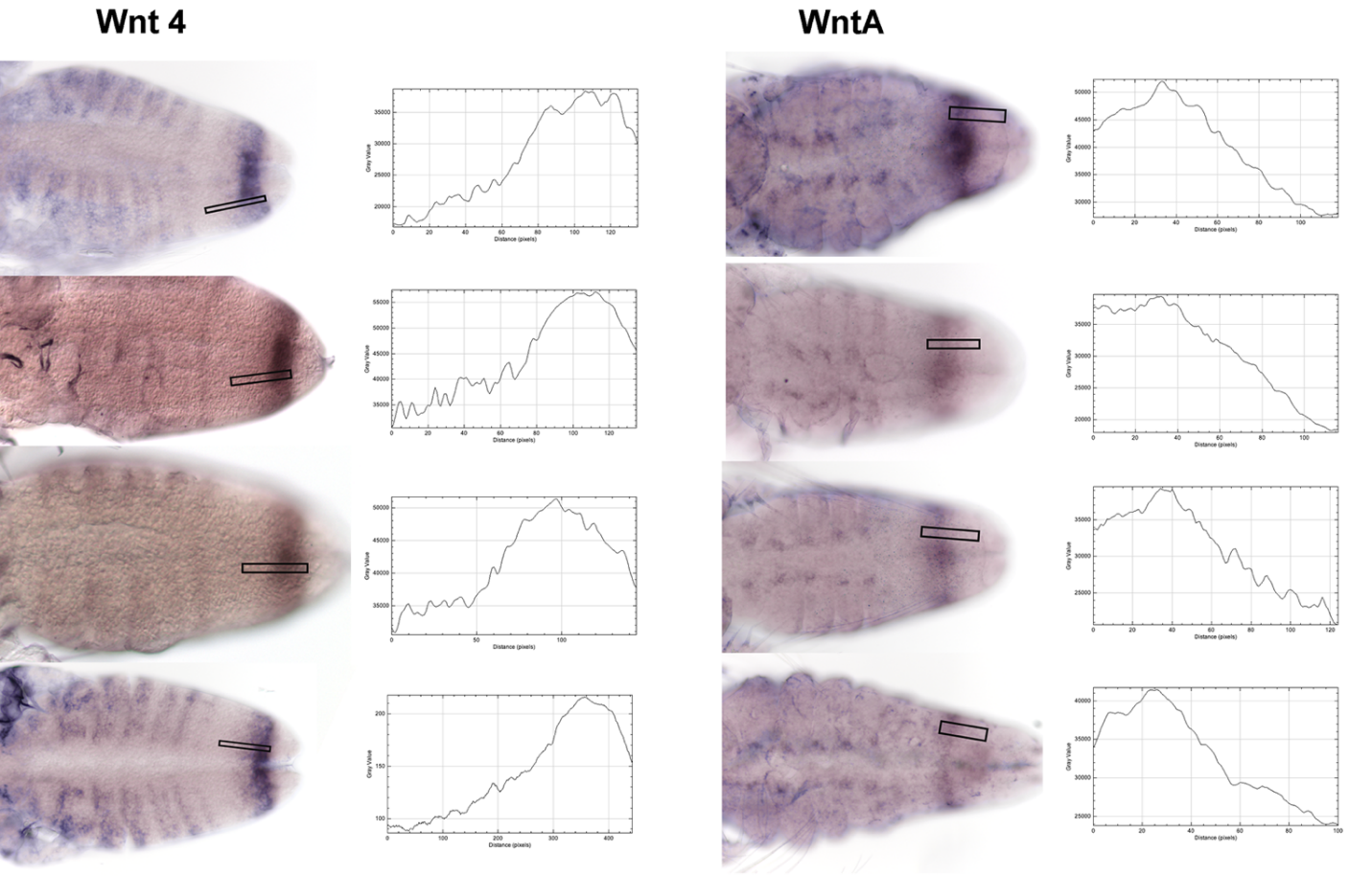

Supplement: Supplementary file 11 — Additional file 11. Seen without the EdU double labeling, both Wnt4 and WntA show graded expression in the posterior growth zone in Thamnocephalus. Expression is quantified using the intensity profile measure in FIJI. [file 13227_2020_147_MOESM11_ESM.docx]
