# Supplementary material for: Elongation during segmentation shows axial variability, low mitotic rates, and synchronized cell cycle domains in the crustacean, Thamnocephalus platyurus
Source: EvoDevo. 2020 Jan 18;11:1. doi: 10.1186/s13227-020-0147-0 (PMC6969478; doi:10.1186/s13227-020-0147-0)

**Additional file 12**. **Comparison of dorsal and ventral cell dynamics in *Thamnocephalus* larvae, visualized by EdU incorporation.** The pattern of Edu and all growth zone measures carry around to the dorsal side of the larvae (shown in focus in A). Focusing through the same specimen shows the normal pattern we describe in the text (B, cells out of focus due to being viewed through dorsal tissue). This corresponding patterning justifies restricting our measures and calculations to the ventral surface since we focus on changes in dimension and other relative features, not absolute measures.


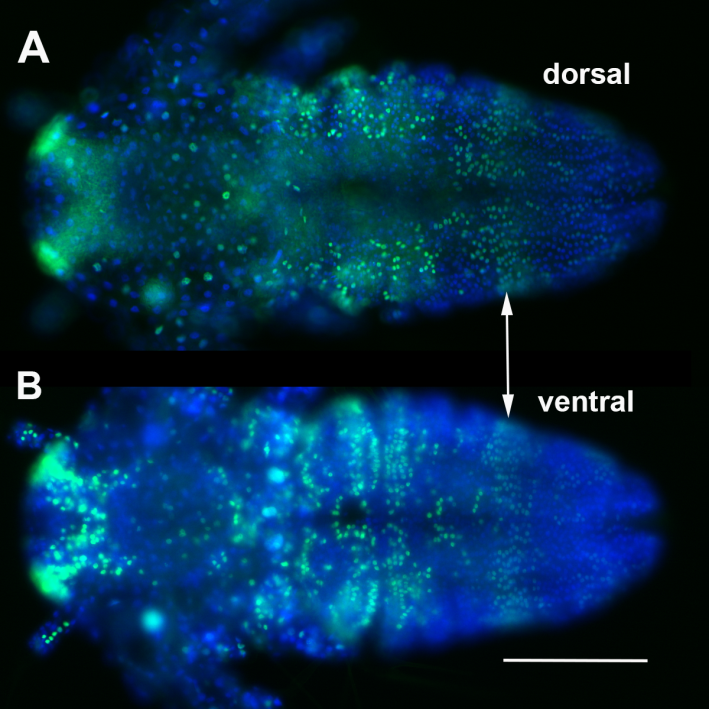

Supplement: Supplementary file 12 — Additional file 12. Comparison of dorsal and ventral cell dynamics in Thamnocephalus larvae, visualized by EdU incorporation. The pattern of Edu and all growth zone measures carry around to the dorsal side of the larvae (shown in focus in A). Focusing through the same specimen shows the normal pattern we describe in the text (B, cells out of focus due to being viewed through dorsal tissue). This corresponding patterning justifies restricting our measures and calculations to the ventral surface since we focus on changes in dimension and other relative features, not absolute measures. [file 13227_2020_147_MOESM12_ESM.docx]
