# Supplementary material for: Elongation during segmentation shows axial variability, low mitotic rates, and synchronized cell cycle domains in the crustacean, Thamnocephalus platyurus
Source: EvoDevo. 2020 Jan 18;11:1. doi: 10.1186/s13227-020-0147-0 (PMC6969478; doi:10.1186/s13227-020-0147-0)

**Additional file 13**. Confocal image of *Thamnocephalus* larva showing the ectodermal projection is a single continuous epithelial layer (E,outside ellipse) underlaid by a mesodermal layer (M, middle ellipse) and the gut (G, interior ellipse)


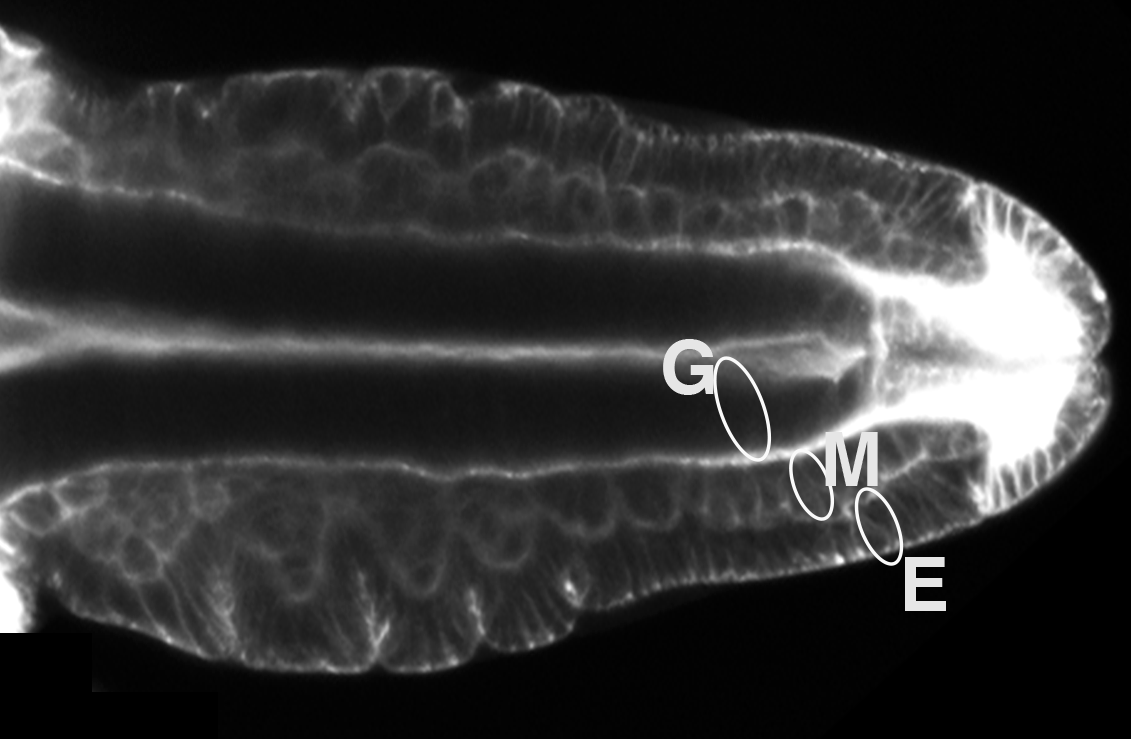

Supplement: Supplementary file 13 — Additional file 13. Confocal image of Thamnocephalus larva showing the ectodermal projection is a single continuous epithelial layer (E, outside ellipse) underlaid by a mesodermal layer (M, middle ellipse) and the gut (G, interior ellipse). [file 13227_2020_147_MOESM13_ESM.docx]
