# Supplementary material for: Elongation during segmentation shows axial variability, low mitotic rates, and synchronized cell cycle domains in the crustacean, Thamnocephalus platyurus
Source: EvoDevo. 2020 Jan 18;11:1. doi: 10.1186/s13227-020-0147-0 (PMC6969478; doi:10.1186/s13227-020-0147-0)

**Additional file 14.** Icons of *Thamnocephalus* trunk region with Engrailed staining illustrating the exact position of measures taken to quantify changes in growth zone dimensions (in blue) corresponding to the measures mapped onto an actual photo.


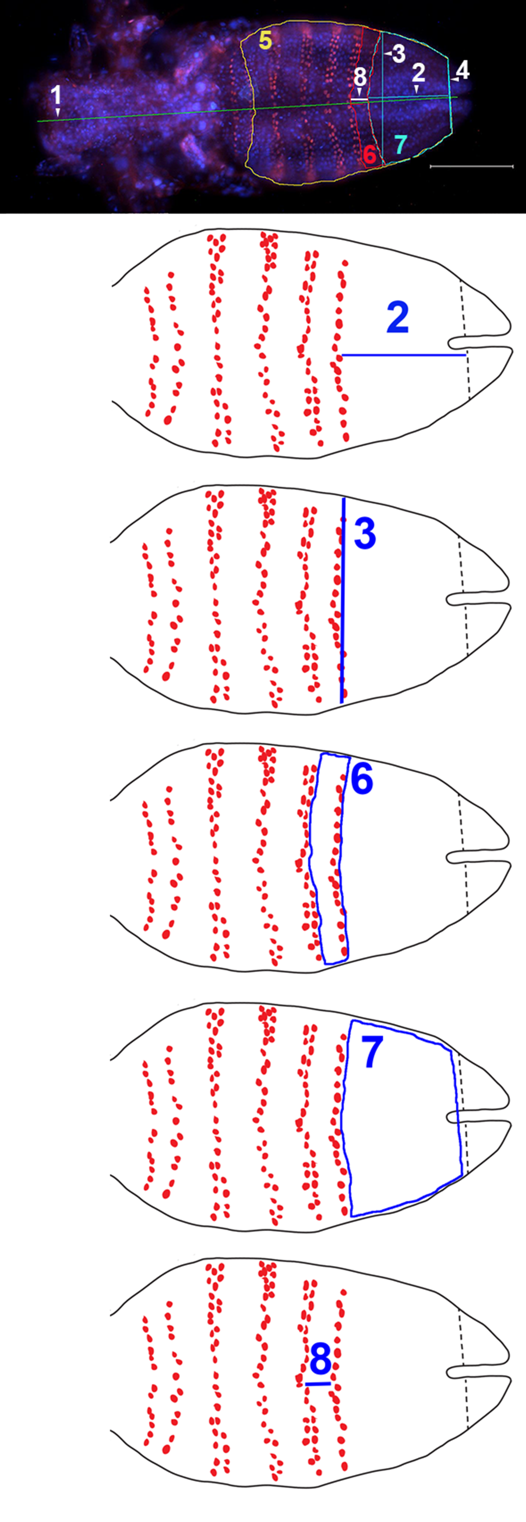

Supplement: Supplementary file 14 — Additional file 14. Icons of Thamnocephalus trunk region with Engrailed staining illustrating the exact position of measures taken to quantify changes in growth zone dimensions (in blue) corresponding to the measures mapped onto an actual photo. [file 13227_2020_147_MOESM14_ESM.docx]
