# Supplementary material for: Elongation during segmentation shows axial variability, low mitotic rates, and synchronized cell cycle domains in the crustacean, Thamnocephalus platyurus
Source: EvoDevo. 2020 Jan 18;11:1. doi: 10.1186/s13227-020-0147-0 (PMC6969478; doi:10.1186/s13227-020-0147-0)
Supplement: Supplementary file 15 — Additional file 15. Top table shows number of larvae scored for each timepoint, with age measured as hours post-hatching. The data were collected by carefully staged timepoints. The bottom table shows those same data subsequently binned according to their developmental age, as indicated by counting the number of Engrailed stripes on the trunk. [file 13227_2020_147_MOESM15_ESM.docx]

**Additional file 15.** Top table shows number of larvae scored for each timepoint, with age measured as hours post-hatching. The data were collected by carefully staged timepoints. The bottom table shows those same data subsequently binned according to their developmental age, as indicated by counting the number of Engrailed stripes on the trunk.

|  | **Age sampled (h post-hatching)** | **Number of larvae scored** |
| --- | --- | --- |
|  | 0 | 25 |
|  | 1 | 26 |
|  | 2 | 14 |
|  | 3 | 36 |
|  | 4 | 18 |
|  | 5 | 26 |
|  | 6 | 23 |
|  | 7 | 28 |
|  | 8 | 21 |
|  | 9 | 24 |
|  | 10 | 26 |
|  | 11 | 20 |
|  | 12 | 23 |
|  | 13 | 20 |
|  | 14 | 30 |
|  | 15 | 20 |
|  | 16 | 15 |
|  | 17 | 19 |
|  | 18 | 19 |
| Total larvae scored |  | 433 |

|  | **Developmental stage (En stripes)** | **Number of larvae** |
| --- | --- | --- |
|  |  |  |
|  | 3 | 24 |
|  | 4 | 26 |
|  | 5 | 18 |
|  | 6 | 50 |
|  | 7 | 24 |
|  | 8 | 29 |
|  | 9 | 45 |
|  | 10 | 37 |
|  | 11 | 44 |
|  | 12 | 35 |
|  | 13 | 28 |
|  | 14 | 22 |
|  | 15 | 34 |
|  | 16 | 11 |
|  | 17 | 6 |
|  |  |  |
| Total larvae scored |  | 433 |
